# NIR-II Emission from Cyclometalated Dinuclear Pt(III) Complexes

**DOI:** 10.1021/acs.inorgchem.3c04314

**Published:** 2024-03-08

**Authors:** Irene Melendo, Sara Fuertes, Antonio Martín, Violeta Sicilia

**Affiliations:** † Instituto de Síntesis Química y Catálisis Homogénea (ISQCH), CSIC–Universidad de Zaragoza, Pedro Cerbuna 12, 50009 Zaragoza, Spain; ‡ Departamento de Química Inorgánica, Escuela de Ingeniería y Arquitectura de Zaragoza, Instituto de Síntesis Química y Catálisis Homogénea (ISQCH), CSIC–Universidad de Zaragoza, Campus Río Ebro, Edificio Torres Quevedo, 50018 Zaragoza, Spain

## Abstract

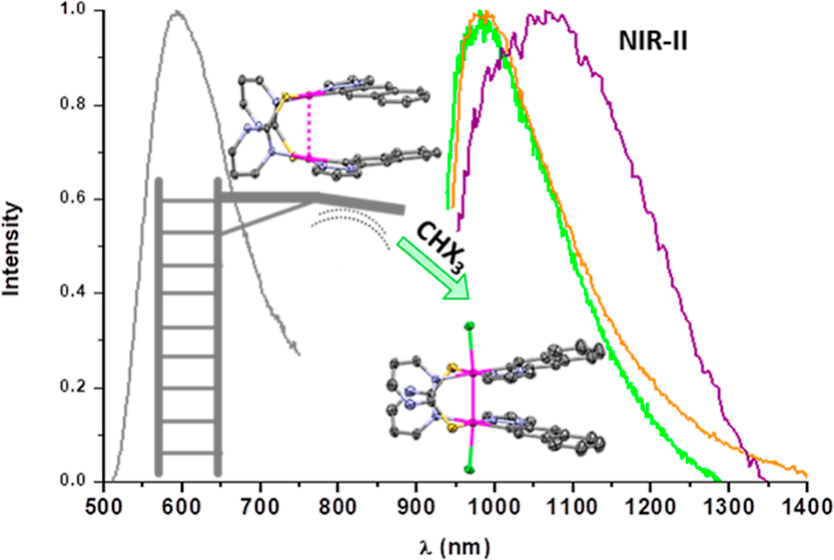

Half-lantern Pt(II) dinuclear complexes [{Pt(C^∧^N_pz_)(μ-S^∧^N^R^)}_2_] (HC^∧^N_pz_ = 1-naphthalen-2-yl-1*H*-pyrazole; R = H, HS^∧^N: 2-mercaptopyrimidine **1**; R = CF_3_, HS^∧^N^F^: 4-(trifluoromethyl)-2-mercaptopyrimidine **2**) were selectively obtained as single isomers with the C^∧^N groups in an *anti*-arrangement and rather short metallophilic interactions (*d*_Pt–Pt_ = 2.8684(2) Å for **2**). They reacted with haloforms in the air and sunlight to obtain the corresponding oxidized diplatinum(III) derivatives [{Pt(C^∧^N_pz_)(μ-S^∧^N^R^)X}_2_] (X = Cl (**1-Cl**), Br (**1-Br**), I (**1-I**, **2-I**)). The single-crystal X-ray structures exhibit Pt–Pt distances typical for the existence of a metal–metal bond, which evidence fairly well the influence of the axial ligand (X). The reactions of **1** and **2** with CHI_3_ in the dark afforded mixtures of [IPt(C^∧^N_pz_)(μ-S^∧^N)_2_Pt(C^∧^N_pz_)CHI_2_] and **1-I** or **2-I**, with the former being the major species under an Ar atmosphere, while the reactions of **1** with CHBr_3_ and CHCl_3_ need light to occur. These Pt_2_(III,III) complexes display low-energy absorptions and emissions that strongly depend on the axial ligand. In the solid state, they show a broad NIR emission ranging from 985 to 1070 nm at RT that suffers a hypsochromic shift when cooling down to 77 K. The photoemissive behavior of the dinuclear Pt(II) and Pt(III) systems is disclosed with the aid of density functional theory calculations.

## Abstract 2

Half-lantern dinuclear Pt(II) complexes can be regarded as an effective springboard to obtain metal−metal bonded Pt_2_(III,III) emitters in the NIR-II spectral window at room temperature.

## Introduction

Near-infrared (NIR) light emitters are drawing increasing attention for their advanced technological applications,^1−4^ in particular, those emitting between 1000 and 1700 nm, formally defined as the NIR-II window. This spectral region, also known as the second transparency or biological window, is typically characterized by a reduced absorption and low photon scattering, enabling a higher degree of penetration through biological tissues and enhancing imaging resolution.^5^ Thus, efficient and long-wavelength NIR light emitting materials with low toxicity and deep penetration are highly attractive for photodynamic therapy,^6^ biomedical sensing,^7^ and optical imaging.^8^ Yet, their design and synthesis are still a challenging endeavor.

Phosphorescent platinum(II) complexes with π-aromatic systems are an active area of research in this field due to the versatile photophysical properties. The square-planar structure gives rise to ground- and excited-state interactions involving self-aggregation and excimer formation, which result in red-to-NIR emissions, typically attributed to metal–metal to ligand charge transfer (MMLCT) [dσ*(Pt)_2_ → π*(L)] or excimer ligand-to-ligand transitions.^9^ However, only a few examples with NIR luminescence close to or beyond 1000 nm at room temperature have been reported to date. Most of them are obtained by molecule stacking of diiminebis(σ-acetylide) Pt(II) derivatives^10−12^ or self-assembled Pt(II) complexes with chelated ligands such as pyrazinyl pyrazolate or pyridyl pyrimidinate.^13^ However, minimal environmental stimuli such as mechanical pressure,^11,14^ temperature,^11,15^ or contact to chemical vapors^10−12,16^ can unintentionally perturb the interactions between the stacked molecules and induce substantial changes in their photophysical properties. To avoid this, the use of 4-bond bridging groups as auxiliary ligands that provide a metal framework with rather short metal–metal distances is a suitable and well-known approach to achieve luminescent compounds with efficient low-lying emissions.^17^ In this regard, half-lantern platinum complexes with C^∧^N cyclometalated ligands and strong intermetallic interactions (*d*_Pt–Pt_ ≤ 3 Å) are some of the most representative cases of ^3^MMLCT [dσ*(Pt)_2_ → π*(C^∧^N)] emitters. Among them, mercapto-^18−31^ or hydroxy-^23,32,33^ substituted N-heterocycles have been typically used as 4-bond bridging ligands to prepare compounds with bright red emissions that, in some cases, have been successfully implemented in organic light-emitting diodes.^24−27,34^ Recently, α-carboline^35^ or 10*H*-pyrido[3,2-*b*][1,4]benzoxazine^36^ has been used instead so as to push further the emission toward the deep red or NIR spectral region because the use of these rigid bridges confine close Pt···Pt contacts. Besides, comprehensive studies on these and also on related complexes have shown that changes on the chromophoric C^∧^N ligands can push the emission to red as well. By these means, not only the lowest unoccupied molecular orbital (LUMO) energy level (π*(C^∧^N)) is modified but also that of the highest occupied molecular orbital (HOMO) (dσ*(Pt)_2_). The π-backdonation from the Pt center to the C^∧^N aromatic system provokes a shortening of the Pt center distance, which leads to a reduced H–L energy gap and consequently a red-shifted emission.^27,32,35−37^

As a result of the short Pt–Pt distances, these lantern-type complexes can experience two-center two-electron oxidations with halogens (X_2_) or halocarbons (RX) to give metal–metal bonded Pt_2_(III,III) complexes.^29,38−43^ These dinuclear Pt(III) species have been considered as nonemissive because of their extremely short-lived dσ* excited states. There have been reported just four d^7^–d^7^ systems with emitting properties: [Pt_2_(C^∧^N)_2_(N^∧^S)_2_Cl_2_] (HS^∧^N = 5-phenyl-1,3,4-oxadiazole-2-thiol; HC^∧^N = 2,4-difluoro-phenylpyridine),^44^ [Pt_2_(HPO_4_)_4_X_2_]^4–^ (X = Cl, Br),^45^ and [Pt_2_(μ-pop)_4_X_2_]^4–^ (pop= (HO_2_P)_2_O, X = Cl, Br, SCN, py)^46^ in the red spectral region and with the latter only being emissive at low temperatures. In addition, compounds [Pt_2_(μ–C_6_H_3_–5-R-2-AsPh_2_)_4_X_2_] (R = Me, ^*i*^Pr; X = Cl, Br, I)^47^ displayed NIR emissions at room and low temperatures.

Throughout our investigations on luminescent half-lantern Pt(II) complexes, we reported the red light-emitting compounds *anti*-[{Pt(bzq)(μ-S^∧^N)}_2_] (Hbzq = benzo[*h*]quinoline, HS^∧^N = 2-mercaptobenzothiazole,^41^ 2-mercaptobenzoxazolate^42^) with photoluminescent quantum yield up to 90% in a solution of toluene. Nonetheless, the analogous compounds [{Pt(bzq)(μ-S^∧^N)}_2_] (HS^∧^N: 2-mercaptopyrimidine,^39^ 4-(trifluoromethyl)-2-mercaptopyrimidine^43^) did not show luminescence in the visible region, nor did their Pt(III) derivatives. In this work, to investigate potential NIR emitters based on dinuclear platinum complexes, we have prepared the half-lantern compounds of Pt(II) [{Pt(C^∧^N_pz_)(μ-S^∧^N^R^)}_2_] (HC∧N_pz_ = 1-naphthalen-2-yl-1*H*-pyrazole; R = H, HS^∧^N: 2-mercaptopyrimidine **1**; R = CF_3_, HS^∧^N^F^: 4-(trifluoromethyl)-2-mercaptopyrimidine **2**). Then, we have explored their redox chemistry by reacting them with haloforms, obtaining the two-electron-oxidized dinuclear Pt(III) complexes [{Pt(C^∧^N_pz_)(μ-S^∧^N^R^)X}_2_] (X = Cl (**1-Cl**), Br (**1-Br**), I (**1-I**, **2-I**)). X-ray diffraction studies, theoretical calculations, and photophysical investigations were carried out on the Pt_2_(II,II) and Pt_2_(III,III) complexes and their results compared to the benzoquinolinate derivatives.

## Experimental Section

### General Methods

Compound [Pt(C^∧^N)Cl(NCMe)](**A**) was prepared accorded to the reported protocol.^48^ 2-Mercaptopyrimidine (HS^∧^N), 4-(trifluoromethyl)-2-mercaptopyrimidine (HS^∧^N^F^), NEt_3_, AgClO_4_, and BaSO_4_ were used as purchased from Across Organics, TCI, Aldrich, and Alfa Aesar, respectively. IR spectra were recorded on a PerkinElmer Spectrum 100 FT-IR Spectrometer (ATR in the range 250–4000 cm^–1^). Mass spectral analyses were performed with a Microflex MALDI-TOF Bruker or an Autoflex III MALDI-TOF Bruker instrument. C, H, and N analyses were carried out in a PerkinElmer 2400 CHNS analyzer. ^1^H, ^19^F, ^195^Pt{^1^H} NMR spectra were recorded on a Bruker Avance 400 MHz instrument using the standard references: SiMe_4_ for ^1^H, CFCl_3_ for ^19^F, and Na_2_PtCl_6_ in D_2_O for ^195^Pt. *J* is given in Hz and assignments are based on ^1^H–^1^H COSY experiments. **Caution!** Perchlorate salts of metal complexes with organic ligands are potentially explosive. Only small amounts of material should be prepared, and these should be handled with great care.

### Preparation of [{Pt(C^∧^N_pz_)(μ-S^∧^N)}_2_] (**1**)

AgClO_4_ (90.0 mg, 0.43 mmol) was added to a suspension of **A** (200.3 mg, 0.43 mmol) in acetonitrile (20 mL). After 7 h of reaction at room temperature in the dark, the mixture was filtered through Celite and washed with acetonitrile. The resultant solution was evaporated to dryness to give a pale-yellow residue, which was then reacted with 2-mercaptopyrimidine (48.4 mg, 0.43 mmol) in 20 mL of acetone/methanol (1/1) and NEt_3_ (0.5 mL) at reflux for 1.5 h. After this time, the suspension was concentrated to ca. 2 mL. The precipitated was filtered, washed with methanol (2 × 3 mL), and dried to give **1** as a yellow solid. Yield: 152.6 mg, 71%. Anal. Calcd for C_34_H_24_N_8_S_2_Pt_2_: C, 40.88; H, 2.42; N, 11.22; S, 6.42. Found: C, 40.42; H, 2.13; N, 10.94; S, 6.56. ^1^H NMR data (400 MHz, CD_2_Cl_2_): 8.77 (dd, ^3^*J*_H6′–H5′_ = 5.7, ^4^*J*_H_6_′–H_4_′_ = 2.6, 1H, H_6_′), 8.32 (dd, ^3^*J*_H4′–H_5_′_ = 4.5, ^4^*J*_H_4_′–H_6_′_ = 2.5, 1H, H_4_′), 7.72 (s, ^3^*J*_Pt–H_ = 56.9, 1H, H_ortho_), [7.58–7.44] (m, 2H, H_naph_), [7.39–7.26] (m, 2H, H_naph_), 7.04 (d, ^3^*J*_H–H_ = 1.8, 1H, H_pz_), [6.86–6.77] (m, 2H, H_pz_, H_5_′), 6.54 (s, 1H, H_meta_), 6.21 (t, ^3^*J*_H–H_ = 2.3, 1H, H_pz_). ^195^Pt{^1^H} NMR (85.6 MHz, THF-*d*_8_): δ = −3549.5 (s). MS (MALDI+): *m*/*z* 997.9 [{Pt(C^∧^N)(μ-S^∧^N)}_2_]^+^.

### Preparation of [{Pt(C^∧^N_pz_)(μ-S^∧^N^F^)}_2_] (**2**)

Compound **2** was synthesized following the same procedure used for **1** but using **A** (250.4 mg, 0.54 mmol), AgClO_4_ (112.3 mg, 0.54 mmol), and (4-trifluoromethyl)-2-mercaptopyrimidine (97.1 mg, 0.54 mmol). **2** was obtained as an orange solid. Yield: 200.5 mg, 65%. Anal. Calcd for C_36_H_22_N_8_S_2_F_6_Pt_2_: C, 38.10; H, 1.95; N, 9.87; S, 5.65. Found: C, 37.79; H, 1.89; N, 10.14; S, 6.06. ^1^H NMR data (400 MHz, acetone-*d*_6_): 9.10 (d, ^3^*J*_H_5_′–H_6_′_ = 5.8, 1H, H_6_′), 7.73 (s, ^3^*J*_Pt–H_ = 54.8, 1H, H_ortho_), 7.57 (d, ^3^*J*_H–H_ = 8.5, 1H, H_naph_), 7.51 (d, ^3^*J*_H–H_ = 7.0, 1H, H_naph_), 7.40 (d, ^3^*J*_H_5_′–H_6_′_ = 5.8, 1H, H_5_′), [7.36–7.28] (m, 3H, 2 H_naph_, H_pz_), 7.24 (d, ^3^*J*_H–H_ = 2.3, 1H, H_pz_), 6.99 (s, 1H, H_meta_), 6.33 (t, ^3^*J*_H–H_ = 2.3, 1H, H_pz_). ^19^F NMR (376.5 MHz, acetone-*d*_6_): – 71.09 (s). ^195^Pt{^1^H} NMR (85.6 MHz, acetone-*d*_6_): δ = −3559.2 (s). MS (MALDI+): *m*/*z* 1134.1 [{Pt(C^∧^N)(μ-S^∧^N^F^)}_2_]^+^

### Preparation of [{Pt(C^∧^N_pz_)(μ-S^∧^N)Cl}_2_] (**1-Cl**)

A suspension of compound **1** (100.1 mg, 0.10 mmol) in CHCl_3_ (85 mL) was left to react in the air, at room temperature, and in sunlight. After 7.5 h of reaction, the mixture was evaporated to dryness and treated with Et_2_O (10 mL); the resulting precipitate was filtered, washed with Et_2_O (10 mL), and dried to give **1-Cl** as a yellow-orange solid. Yield: 77.2 mg, 72%. Anal. Calcd for C_34_H_24_Cl_2_N_8_S_2_Pt_2_: C, 38.17; H, 2.26; N, 10.47; S, 5.99. Found: C, 37.73; H, 2.19; N, 10.08; S, 5.57. ^1^H NMR data (400 MHz, CD_2_Cl_2_): δ = 9.50 (dd, ^3^*J*_H_6_′–H_5_′_ = 5.8, ^4^*J*_H_6_′–H_4_′_ = 2.3, 1H, H_6_′), 8.58 (dd, ^3^*J*_H_4_′–H_5_′_ = 4.6, ^4^J_H_4_′–H_6_′_ = 2.3, 1H, H_4_′), [7.63–7.58] (m, 1H, H_naph_), 7.53 (d, ^3^*J*_H–H_ = 2.2, 1H, H_pz_), [7.48–7.38] (m, 3H, H_naph_), 7.32 (s, ^3^*J*_Pt–H_ = 39.0, 1H, H_ortho_), [7.05–6.99] (m, 2H, H_5_′, H_pz_), 6.58 (s, 1H, H_meta_), 6.54 (t, ^3^*J*_H–H_ = 2.6, 1H, H_pz_). ^195^Pt{^1^H} NMR (85.6 MHz, CD_2_Cl_2_): δ = −2368.1 (s). MS (MALDI+): *m*/*z* 1033.8 [{Pt(C^∧^N)(μ-S^∧^N)}_2_Cl]^+^.

### Preparation of [{Pt(C^∧^N_pz_)(μ-S^∧^N)Br}_2_] (**1-Br**)

CHBr_3_ (25 μL, 0.28 mmol) was added to a suspension of **1** (70.8 mg, 0.071 mmol) in acetone (90 mL) in the air, at RT, and in sunlight. After 7 h of reaction, the suspension was concentrated to ca. 3 mL, treated with *n*-hexane (20 mL), and then filtered and washed to give **1-Br** as a dark orange solid. Yield: 58.8 mg, 72%. Anal. Calcd for C_34_H_24_Br_2_N_8_S_2_Pt_2_: C, 35.24; H, 2.09; N, 9.67; S, 5.53. Found: C, 34.79; H, 1.96; N, 9.48; S, 5.47. ^1^H NMR data (400 MHz, CD_2_Cl_2_): δ = 9.63 (dd, ^3^*J*_H_6_′–H_5_′_ = 5.9, ^4^*J*_H_6_′–H_4_′_ = 2.3, 1H, H_6_′), 8.56 (dd, ^3^*J*_H_4_′–H_5_′_ = 4.5, ^4^J_H_4_′–H_6_′_ = 2.3, 1H, H_4_′), [7.62–7.57] (m, 1H, H_naph_), 7.55 (d, ^3^*J*_H–H_ = 2.0, 1H, H_pz_), [7.46–7.39] (m, 3H, H_naph_), 7.26 (s, ^3^*J*_Pt–H_ = 38.6, 1H, H_ortho_), [7.04–6.99] (m, 2H, H_5_′, H_pz_), 6.57 (t, ^3^*J*_H–H_ = 2.6, 1H, H_pz_), 6.51 (s, 1H, H_meta_). ^195^Pt{^1^H} NMR (85.6 MHz, CD_2_Cl_2_): δ = −2516.1 (s). MS (MALDI+): *m*/*z* 1078.7 [{Pt(C^∧^N)(μ-S^∧^N)}_2_Br]^+^.

### Preparation of [{Pt(C^∧^N_pz_)(μ-S^∧^N)I}_2_] (**1-I**)

CHI_3_ (103.0 mg, 0.26 mmol) was added to a suspension of **1** (65.1 mg, 0.065 mmol) in acetone (15 mL) in the air, at RT, and in sunlight. After 16 h of reaction, the suspension was concentrated to ca. 2 mL, and then 20 mL of *n*-hexane was added to give a red garnet solid that was filtered, washed, and dried. Yield: 66.0 mg, 81%. Anal. Calcd for C_34_H_24_I_2_N_8_S_2_Pt_2_: C, 32.60; H, 1.93; N, 8.95; S, 5.12. Found: C, 32.15; H, 2.10; N, 8.52; S, 4.90. ^1^H NMR data (400 MHz, CD_2_Cl_2_): δ = 9.82 (dd, ^3^*J*_H_6_′–H_5_′_ = 5.8, ^4^*J*_H_6_′–H_4_′_ = 2.3, 1H, H_6_′), 8.53 (dd, ^3^*J*_H_4_′–H_5_′_ = 4.4, ^4^J_H_4_′–H_6_′_ = 2.3, 1H, H_4_′), [7.61–7.55] (m, 2H, H_naph_, H_pz_), [7.44–7.39] (m, 3H, H_naph_), 7.18 (s, ^3^*J*_Pt–H_ = 40.7, 1H, H_ortho_), 7.03 (d, ^3^*J*_H–H_ = 3.3, 1H, H_pz_), 6.99 (dd, ^3^*J*_H_5_′–H_6_′_ = 5.8, ^3^*J*_H_5_′–H_4_′_ = 4.6, 1H, H_5_′), 6.60 (t, ^3^*J*_H–H_ = 2.6, 1H, H_pz_), 6.42 (s, 1H, H_meta_). ^195^Pt{^1^H} NMR (85.6 MHz, CD_2_Cl_2_): δ = −2767.5 (s). MS (MALDI+): *m*/*z* 1124.7 [{Pt(C^∧^N)(μ-S^∧^N)}_2_I]^+^.

### Preparation of [{Pt(C^∧^N_pz_)(μ-S^∧^N^F^)I}_2_] (**2-I**)

Compound **2-I** was synthesized by the same procedure as that of **1-I** but using **2** (70.2 mg, 0.062 mmol) and CHI_3_ (97.3 mg, 0.25 mmol). **2-I** was obtained as a purple solid. Yield: 66.8 mg, 78%. Anal. Calcd For C_36_H_22_F_6_I_2_N_8_S_2_Pt_2_: C, 31.14; H, 1.60; N, 8.07; S, 4.62. Found: C, 30.79; H, 1.54; N, 7.96; S, 4.45. ^1^H NMR data (400 MHz, acetone-*d*_6_): δ = 10.14 (d, ^3^*J*_H_5_′–H_6_′_ = 6.0, 1H, H_6_′), 7.83 (d, ^3^*J*_H–H_ = 2.3, 1H, H_pz_), 7.66 (d, ^3^*J*_H_5_′–H_6_′_ = 6.0, 1H, H_5_′), [7.64–7.58] (m, 2H, H_naph_, H_pz_), [7.53–7.47] (m, 1H, H_naph_), [7.46–7.38] (m, 2H, H_naph_), 7.24 (s, ^3^*J*_Pt–H_ = 40.7, 1H, H_ortho_), 6.92 (s, 1H, H_meta_), 6.73 (t, ^3^J_H–H_ = 2.6, 1H, H_pz_). ^19^F NMR (376.5 MHz, acetone-*d*_6_): – 70.91 (s). ^195^Pt{^1^H} NMR (85.6 MHz, acetone-*d*_6_): δ = −2763.3 (s). MS (MALDI+): *m*/*z* 1261.1 [{Pt(C^∧^N)(μ-S^∧^N^F^)I}_2_]^+^.

### Computational Methods

Density functional calculations were carried out on the ground (S_0_) and triplet (T_1_) state with the Gaussian 09 suite of programs, using the M06 hybrid density functional^49^ together with Grimme’s D3 dispersion correction.^50^ The ECP-60-mwb for Pt and ECP-46-mwb, for I, pseudopotentials^51^ were used, and the 6-31G(d)^52,53^ basis sets were used for all other atoms. General geometry optimizations were performed without any symmetry restriction and in the gas phase. Frequency calculations were performed in order to determine the nature of the stationary points found in S_0_ and T_1_ (no imaginary frequencies for minima). The time-dependent density-functional (TD-DFT) calculations were also carried out in the gas phase. Mulliken population analysis was carried out as implemented in the Gaussian 09 package.^54^ The ChemissianLab program package was used for analysis and graphic representation of molecular orbitals and for Mayer bond order (BO) analysis. Atomic coordinates for the optimized structures are included as a separate .xyz file.

## Results and Discussion

### Synthesis and Characterization of New Pt_2_(II,II) and Pt_2_(III,III) Complexes. Reactivity of Pt_2_(II,II) Half-Lantern Compounds toward Haloforms

**Scheme 1:**
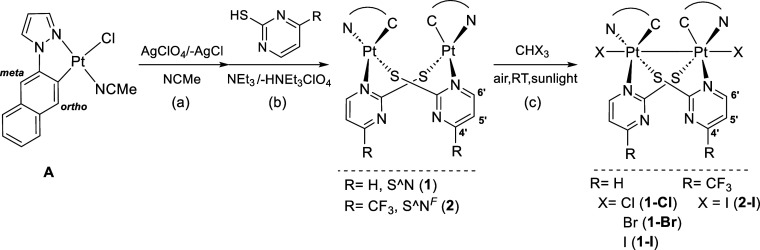
Synthesis Route for Dinuclear Pt(II) and Pt(III) Complexes Numerical scheme for NMR characterization.

Compounds **1** and **2** were obtained following the synthetic pathway depicted in Scheme 1, which starts with the chlorine abstraction from compound [Pt(C^∧^N_pz_)Cl(NCMe)] (HC^∧^N = 1-naphthalen-2-yl-1*H*-pyrazole, **A**) with AgClO_4_ in acetonitrile (step a) followed by the elimination of AgCl and evaporation of the solvent. Afterward, the residue was treated with equimolecular amounts of HS^∧^N^R^ and excess of NEt_3_ in refluxing acetone/methanol for 1.5 h (step b). The workup of the reactions afforded the corresponding compounds [{Pt(C^∧^N_pz_)(μ-S^∧^N^R^)}_2_] (R = H, HS^∧^N: 2-mercaptopyrimidine **1**; R = CF_3_, HS^∧^N^F^: 4-(trifluoromethyl)-2-mercaptopyrimidine **2**) as pure yellow and orange solids in good yields.

**Figure 1:**
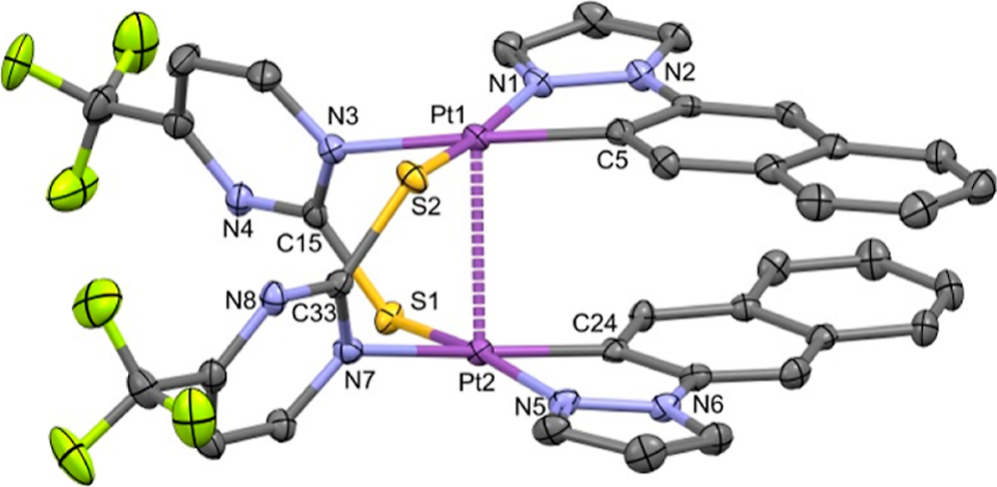
Molecular structure of **2**. Thermal ellipsoids are drawn at their 50% probability level; solvent molecules and hydrogens are omitted for clarity.

These compounds were fully characterized through different techniques (see the Experimental Section). The ^1^H and ^19^F NMR spectra (Figures S1–S2) display the expected set of signals for a single symmetric isomer, the anti one, as confirmed by single-crystal X-ray diffraction of complex **2** (see Figure 1 and Table 1). As can be seen, complex [{Pt(C^∧^N_pz_)(μ-S^∧^N^F^)}_2_] (**2**) is a neutral dinuclear species of Pt(II) formed by two fragments “Pt(C^∧^N_pz_)” doubly bridged by two 4-(trifluoromethyl)-2-mercaptopyrimidine ligands. They show an anti configuration and a Pt···Pt distance of 2.8684(2) Å, which is among the shortest reported for half-lantern diplatinum(II) complexes.^20,24,26,28,30,41,55,56^ Each Pt(II) center coordinates to the two donor atoms of the naphthyl pyrazole ligand (C, N), a N atom of one mercaptopyrimidine group (S^∧^N^F^), and a S atom of the other one. Each Pt(II) center shows a distorted square-planar environment, which is mainly due to the small bite angle of the *C*,*N*-cyclometalated ligand [81.1(13)° Pt(1), 80.9(1)° Pt(2)]. This isomer has a head-to-tail configuration of the two bridging S∧N^F^ groups with a 2-fold axis perpendicular to the midpoint of the Pt(II)···Pt(II) line. Regarding the C^∧^N_pz_ groups, an *anti*-arrangement of these can be found within the complex.

**Table 1:** Selected Bond Lengths (Å) and Angles (deg) of Complex **2**

distances (Å)	Pt1	Pt2
Pt1–Pt2	2.8684 (2)	
Pt–N_C∧N_	2.026 (3)	2.022 (3)
Pt–C_C∧N_	2.002 (4)	2.001 (4)
Pt–N_N∧S_	2.137 (3)	2.131 (3)
Pt–S_N∧S__′_	2.2726 (9)	2.2787 (9)
Angles (deg)		
N_C∧N_–Pt–C_C∧N_	81.10 (13)	80.95 (13)
C_C∧N_–Pt–S_N∧S′_	95.77 (11)	95.88 (10)
S_N∧S′_–Pt–N_N∧S_	92.69 (9)	92.34 (8)
N_C∧N_–Pt–N_N∧S_	91.03 (12)	91.35 (12)

Also, they display an offset stacking [torsion angle N(1)–Pt(1)–Pt(2)–N(5), 120.2(2)°] without close π–π intramolecular interactions, most likely to reduce repulsions in this kind of compounds.^28,55^ The platinum coordination planes are not completely parallel to one another since the interplanar angle is 15.12(8)°. Nonetheless, the short Pt···Pt distance and the perpendicularity between the Pt–Pt line and both metal coordination planes [angles = 8.64(6)° Pt(1); 7.85(5)° Pt(2)] suggest a significant interaction of the 5d_*z*^2^_ orbitals of both platinum centers. Within the crystal packing, weak π–π intermolecular interactions between the C^∧^N ligands (C–C distances between 3.34 and 3.40 Å) give rise to slipped π–π stacking of adjacent molecules (Figure S3).

**Figure 2:**
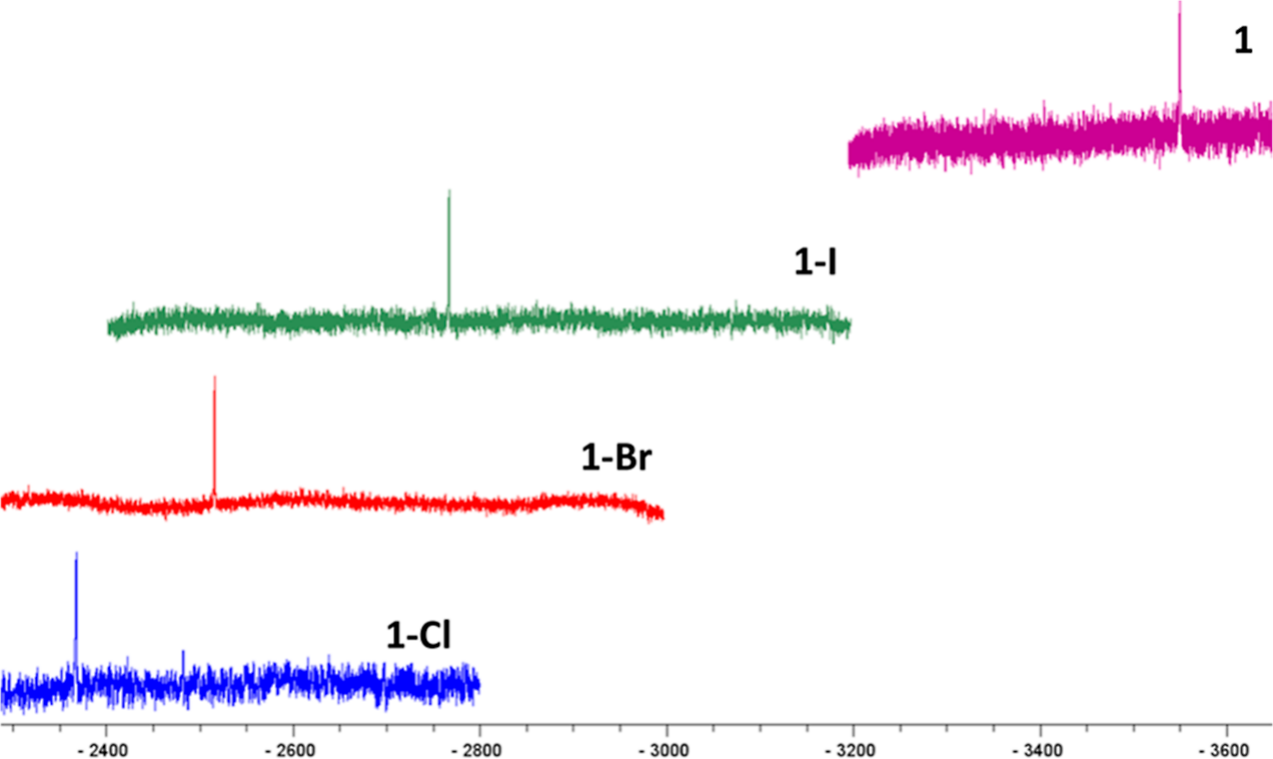
^195^Pt{^1^H} NMR spectra of **1**, **1-Cl**, **1-Br**, and **1-I**.

**Figure 3:**
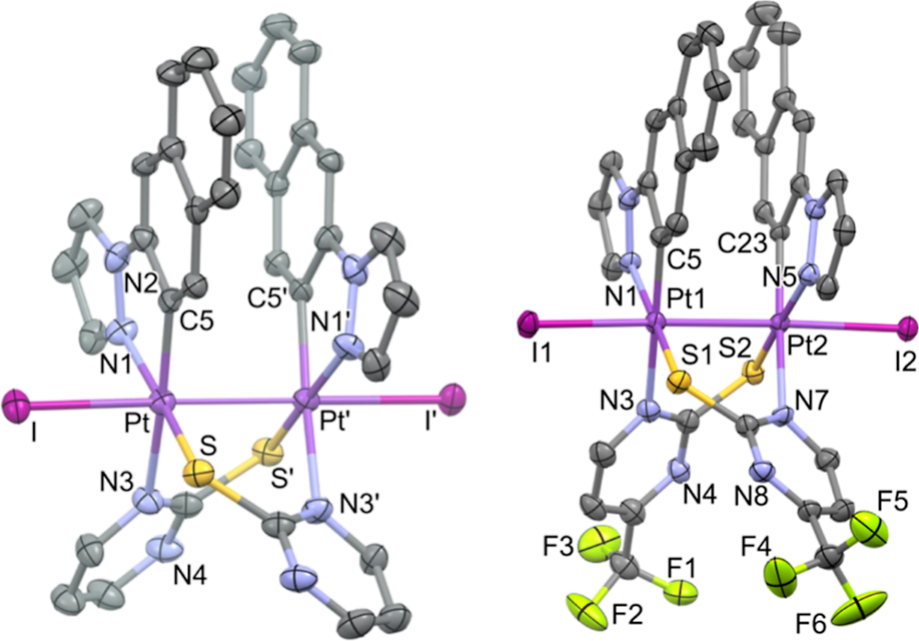
Molecular structure of **1-I** (left) and **2-I** (right). Thermal ellipsoids are drawn at their 50% probability level; solvent molecules and hydrogens are omitted for clarity.

As expected from the short Pt–Pt distance and as found in other lantern,^57^ half-lantern,^29,39^ or even pyrazolate^58,59^ dinuclear Pt(II) complexes, compound **1** undergoes two-center two-electron oxidation upon treatment with haloforms CHX_3_ (X = Cl, Br, and I) in the air and sunlight to give the corresponding dihalogenated diplatinum(III) complexes [{Pt(C^∧^N_pz_)(μ-S^∧^N)X}_2_] (X = Cl **1-Cl**, Br **1-Br**, I **1-I**) as yellow-orange, orange, and red garnet solids, respectively, in high yield (see Scheme 1 path c). For comparison and reproducibility, compound **2-I** was prepared by the reaction of **2** with CHI_3_ in the air and sunlight. All compounds were fully characterized (see the Experimental Section and Figures 2, 3 and S4–S9 in the Supporting Information). Their ^1^H NMR spectra are very similar and show small changes with respect to the corresponding starting complexes. See the downfield [0.7–1.1 ppm] and upfield [∼0.5 ppm] shifts of the signals corresponding to H_6_′ and H_ortho_ to the Pt–C bonds. Additionally, the ^3^*J*_Pt–_H_ortho_ values decrease in comparison with those found in their parent compounds due to the higher oxidation state of the Pt center. This was also evident from the significant downfield shift (ΔδPt: 782–1181 ppm) of their ^195^Pt NMR signals from **1** and **2** (δ^195^Pt ∼ −3550 ppm) (see Figures 2 and S8). Besides, the ^195^Pt resonances appear more deshielded as the electronegativity of the axial ligand is greater (−2368.1 (**1-Cl**) vs −2767.5 ppm (**1-I**)), as in other reported Pt_2_(III,III).^52^

The X-ray structures of **1-Cl**, **1-I**, and **2-I** are depicted in Figures 3 and S9, and a selection of bond distances and angles is listed in Table 2. They confirmed the anti configuration of the molecule along with the retention of the half-lantern structure with respect to the starting complexes.

**Table 2:** Selected Bond Lengths (Å) and Angles (deg) of Complexes **1-Cl**, **1-I**, and **2-I**

distances (Å)	1-Cl	1-I	2-I (Pt1)	2-I (Pt2)
Pt–Pt	2.5898 (6)	2.6365 (5)	2.6491 (2)	
Pt–N_C∧N_	2.062 (7)	2.061 (7)	2.048 (4)	2.053 (4)
Pt–C_C∧N_	2.009 (8)	2.029 (7)	2.019 (5)	2.022 (4)
Pt–N_S∧N_	2.139 (7)	2.168 (6)	2.172 (4)	2.166 (4)
Pt–S_S∧N_	2.307 (2)	2.292 (2)	2.2913 (11)	2.2932 (12)
Pt–X	2.4428 (19)	2.7518 (5)	2.7678 (3)	2.7632 (3)
Angles (deg)				
N_C∧N_–Pt–C_C∧N_	79.9 (3)	80.6 (3)	80.40 (17)	80.48 (17)
N_C∧N_–Pt–N_S∧N_	93.6 (3)	90.8 (3)	95.17 (15)	93.11 (15)
C_C∧N_–Pt–S_S∧N_	97.7 (2)	95.2 (2)	94.67 (13)	95.79 (14)
N_S∧N_–Pt–S_S∧N_	88.87 (19)	93.37 (19)	89.80 (11)	90.62 (11)
C_C∧N_–Pt–X	88.8 (2)	85.5 (2)	85.99 (13)	86.46 (12)
N_C∧N_–Pt–X	88.30 (17)	87.58 (18)	89.49 (10)	87.16 (10)
N_S∧N_–Pt–X	90.96 (17)	94.71 (18)	94.92 (10)	93.44 (10)
S_S∧N_–Pt–X	89.04 (7)	87.69 (5)	87.23 (3)	88.87 (3)
Pt–Pt–X	175.00 (5)	174.92 (2)	175.476 (10)	176.968 (11)

Each Pt(III) center has a distorted octahedral environment with the axial positions occupied by a halogen atom (Cl or I) and the other Pt(III) center and with the X–Pt–Pt angles being close to 175°. The Pt–Pt distances (2.5898(6) Å **1-Cl**, 2.6365(5) Å **1-I**, 2.6491(2) Å **2-I**) are shorter than that found for complex **2**, indicating the existence of a single metallic bond between both Pt(III) centers. They also reflect the trans influence of the axial ligand X, with that of the chloride derivative being shorter than the iodide one.^41,42,47,59^ All intermetallic distances fall in the low range of those observed for Pt_2_(III,III) complexes^18,29,41,42^ and they are even shorter than the bzq derivatives with the same bridging ligand [{Pt(bzq)(μ-S^∧^N)X}_2_] (X: Cl 2.6132(2) Å, I 2.6401(2) Å).^39^ The two Pt coordination planes are almost parallel to each other with small interplanar angles [11.10(5)° **1-Cl**, 9.32(7)° **1-I**, 8.90(10)° **2-I**] and Pt–Pt lines near perpendicular to the coordination planes (4.12(6)° for **2-I**). Inspection of the crystal packing revealed some π–π interactions for **1-Cl** between the C^∧^N groups of adjacent molecules (d_C–C_: 3.333 Å), stacking them into infinite 1D-chains (see Figure S9). This arrangement is also supported by H···Cl interactions (*d*_H–Cl_: 2.821 Å, *d*_C–Cl_: 3.699 Å) between the C^∧^N and the axial Cl ligands from side-on molecules.

**Scheme 2:**
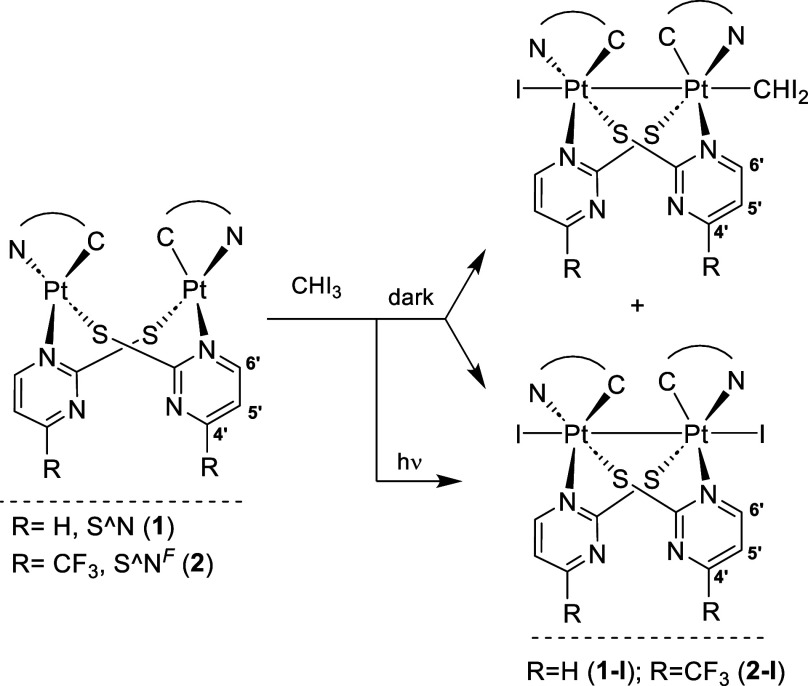
Oxidation Reactions of **1** and **2** with CHI_3_

Concerning the reactivity of **1** and **2** toward haloforms, there are some particularities worth mentioning. In the first place, the reactions of **1** with CHCl_3_ and CHBr_3_ only proceed with sunlight. If the reaction is performed in the dark, overnight, we recover the starting material (see Figure S10 as an example of **1** with CHBr_3_). Second, the reactions of **1** and **2** with CHI_3_ can take place in the air and protected from light, but it gives mixtures of two species [IPt(C^∧^N_pz_)(μ-S^∧^N)_2_Pt(C^∧^N_pz_)CHI_2_] and [{Pt(C^∧^N_pz_)(μ-S^∧^N)I}_2_] (**1-I** or **2-I**) (see Scheme 2 and Figure S11). They were detected by NMR spectroscopy; the former one presents two sets of signals for the C^∧^N_pz_ and the N^∧^S ligands due to the nonequivalence of the two Pt moieties and a singlet with ^195^Pt satellites corresponding to the CHI_2_ fragment [δ_H_: 4.40, ^2^*J*_Pt,H_ = 20.8 Hz (R = H); 4.42, ^2^*J*_Pt,H_ = 19.4 Hz (R = CF_3_)]. Then, the simultaneous formation of both species, [IPt(C^∧^N_pz_)(μ-S^∧^N)_2_Pt(C^∧^N_pz_)CHI_2_] and [{Pt(C^∧^N_pz_)(μ-S^∧^N)I}_2_], in the dark points to a radical mechanism for the thermal oxidation.^60^

To confirm this, we carried out comparative ^1^H NMR experiments of **1** with CHI_3_ (1:4) in acetone-*d*_6_, protected from light, under argon and oxygen atmospheres. As shown in Figure S12, after 15 min (*t*_0_), the reaction was completed giving mixtures of **1-I** and [IPt(C^∧^N_pz_)(μ-S^∧^N)_2_Pt(C^∧^N_pz_)CHI_2_] with different ratios depending on the experimental conditions. Under an Ar atmosphere, the relation between compounds **1-I** and [IPt(C^∧^N_pz_)(μ-S^∧^N)_2_Pt(C^∧^N_pz_)CHI_2_] was 1:2.85; however in an O_2_ atmosphere, it was 1:0.28. Also, we observed that the mixture **1-I** and [IPt(C^∧^N_pz_)(μ-S^∧^N)_2_Pt(C^∧^N_pz_)CHI_2_] remains unchanged for at least 24 h while the reaction was kept in the dark (see Figure S12). Nonetheless, if the reactions with CHI_3_ are performed in a straightforward manner in the air and in sunlight, compounds **1** and **2** are completely reacted to yield the corresponding complexes: [{Pt(C^∧^N_pz_)(μ-S^∧^N^R^)I}_2_] (R = H **1-I**, CF_3_**2-I**). Thus, the experimental results are closely related to our previous research, in which detailed mechanistic studies were performed for the activation of C–X bonds in haloforms by dinuclear Pt(II) complexes.^39,59^ In short, these reactions proceed through a radical pathway (eqs 1–4) through the thermal or photochemical homolytic cleavage of the X–C bond in a [Pt···Pt···XCHX_2_] adduct.
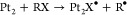
1
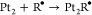
2
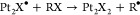
3
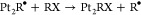
4

**Scheme 3:**
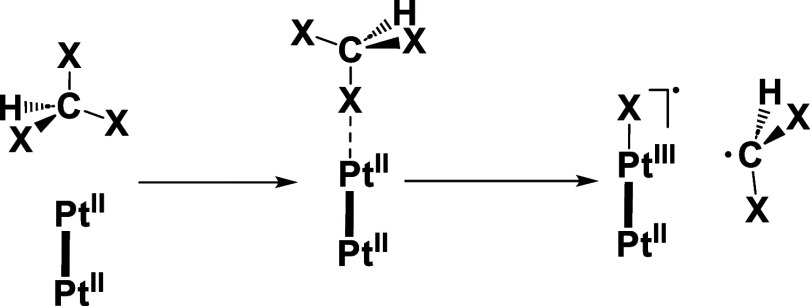
Proposed Pathway for the First Step in the Conversion of Pt(II,II) into Pt(III,III) Complexes by Reaction with Haloforms

The concomitant formation of Pt_2_X^•^ and ^•^CHX_2_ (R^•^) radicals justifies the simultaneous formation of Pt_2_X_2_ and Pt_2_RX, with O_2_ acting as an efficient radical trap (R^•^), increasing the ratio [{Pt(C^∧^N)(μ-S^∧^N)I}_2_]:[IPt(C^∧^N)(μ-S^∧^N)_2_Pt(C^∧^N)CHI_2_]. Also, in the presence of CHI_3_, species [IPt(C^∧^N)(μ-S^∧^N)_2_Pt(C^∧^N)CHI_2_] transform completely into [{Pt(C^∧^N)(μ-S^∧^N)I}_2_] under irradiation with UV-light or sunlight. In this case, the reaction of **1** with CHI_3_ is thermally initiated, while those with CHBr_3_ and CHCl_3_ need sunlight to occur. By contrast, the reactions of the analogous [{Pt(bzq)(μ-S^∧^N)}_2_] (HN^∧^S = 2-mercaptopyrimidine)^39^ with both CHI_3_ and CHBr_3_ proceeds in the dark, indicating the significance of the C^∧^N group in this reactivity. In previous works,^39,59^ we demonstrated that MMLCT species, with the HOMO being a dσ* orbital constructed from d_*z*^2^_ Pt atoms, in their ground or excited state, are those that trigger the C–X activation (see Scheme 3).

Therefore, both the bond dissociation energies (increasing in the sequence C–I < C–Br < C–Cl) and the energy of the HOMO in the [Pt_2_] complex are crucial in the first step of this reaction. Since the π-backdonation from the Pt center to the naph-pz system in **1** is expected to be minor in relation to the bzq one, due to a less extended aromatic system, a weaker overlapping of the d_*z*^2^_ Pt orbitals would be found in **1**. To check this, we carried out DFT calculations on complexes **1** and [{Pt(bzq)(μ-S^∧^N)}_2_] (HN^∧^S = 2-mercaptopyrimidine), finding a decrease of the σ*(d_*z*^2^_–d_*z*^2^_) HOMO energy in **1** (−5.10 eV) when compared to that of [{Pt(bzq)(μ-S^∧^N)}_2_] (−4.99 eV, see Figure S13) due to a smaller energy splitting of the bonding σ and antibonding σ* orbitals. Then, there is need for light to reach MMLCT excited species in **1** to promote the C–Br bond breaking in the first step of the radical process.

### Photophysical and Computational Studies

#### Absorption Spectra and DFT Studies

**Figure 4:**
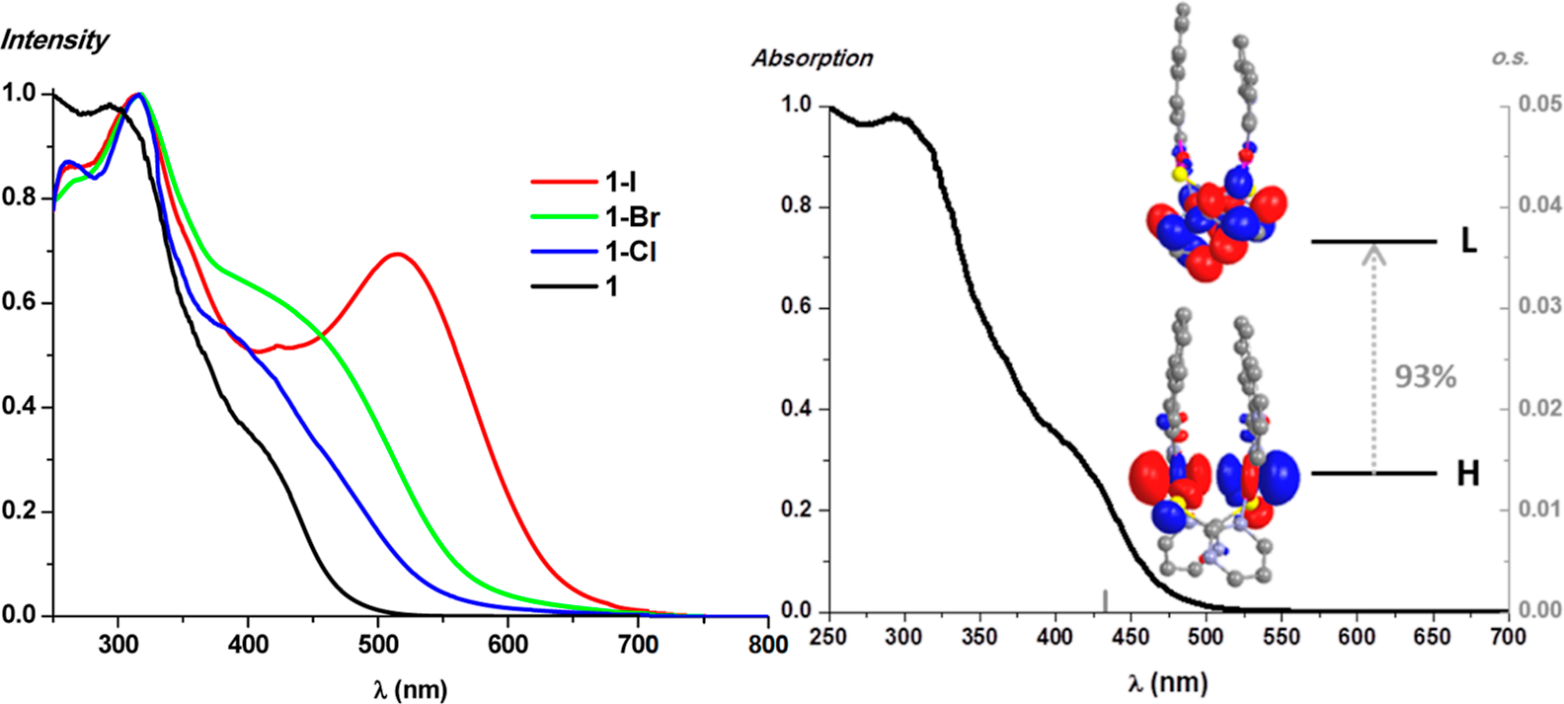
Left: normalized diffuse reflectance spectra in the solid state. Right: normalized absorption spectra in the solid state of **1**, S_1_ calculated transitions in the gas phase (bar) and molecular orbital plots (isovalue 0.03).

The UV–vis absorption spectra of the Pt_2_(II,II) and Pt_2_(III,III) complexes were measured in solution and in the solid state (Figures 4 and S14 and Table 3). The Pt_2_(II,II) complexes in the solid state show some weak low energy absorptions at ca. 415 nm, while those for the Pt_2_(III,III) derivatives appear at higher wavelengths, λ > 450 nm (see Figure 4 left for **1** and **1-X**), indicating a noticeable change in the frontier orbitals. In the latter, the absorptions are strongly dependent on the axial ligand X, with the energy maxima decreasing in the order Cl^–^ > Br^–^ > I^–^. In the solution of CH_2_Cl_2_, we observe these same trends (see Figure S14 top). The S^∧^N^F^ compounds (**2** and **2-I**) present a greater solubility than their S^∧^N counterparts. Thus, they could be measured in different solvents (10^–4^ M), showing a small negative solvatochromism, which is characteristic of charge transfer transitions (Figure S14 bottom). As reported in the literature, the low energy bands in the Pt_2_(II,II) half-lantern complexes have been typically attributed to a ^1^MMLCT [dσ*(Pt–Pt) → π*(C^∧^N)].^18−30,32,33,35,36^ Those for metal–metal bond Pt_2_(III,III) derivatives are originated from an admixture of axial ligand (X) to metal–metal charge transfer, (^1^XMMCT) and ^1^MC [dσ–dσ*] transitions^47,61^ or from ^1^LMMCT [π(S^∧^N) → dσ*(Pt–Pt)] transitions.^44^

**Table 3:** Absorption Data in Solution (10^–4^ M) and in the Solid State^a^ at 298 K

compound	λ/nm (10^3^ ε M^–^^1^ cm^–^^1^)
**1**	286 (45.9), 315_sh_ (31.2), 357_sh_ (10.9), 405(4.7) **CH**_**2**_**Cl**_**2**_
	250, 305, 364, 415 **solid**
**2**	313 (23.2), 345_sh_ (10.6) **THF**
	318 (31.0), 362 (8.8), 370_sh_ (7.9), 404 (4.7) **CH**_**2**_**Cl**_**2**_
	323 (28.9), 362 (9.5), 391_sh_ (6.3) **MeCN**
	295, 420 tail to 525 **solid**
**1-Cl**	300_sh_ (36.4), 345 (11.1), 371_sh_ (7.0), 412 (4.5) **CH**_**2**_**Cl**_**2**_
	259, 316, 384, 454 tail to 625 **solid**
**1-Br**	327 (25.8), 344_sh_ (20.7), 378 (12.8), 429 (8.8) **CH**_**2**_**Cl**_**2**_
	268, 317, 460 tail to 650 **solid**
**1-I**	312_sh_ (37.1), 351 (18.4), 440_sh_ (11.1), 501 (18.5) **CH**_**2**_**Cl**_**2**_
	262, 316, 353, 423, 514 tail to 700 **solid**
**2-I**	312_sh_ (37.7), 372 (15.0), 510 (20.8) **toluene**
	318_sh_ (33.6), 368 (17.1), 506 (22.9) **CH**_**2**_**Cl**_**2**_
	312_sh_ (33.4), 370 (15.4), 504 (20.4) **THF**
	313_sh_ (34.5), 365 (17.7), 500 (19.7) **MeCN**
	262, 307, 379, 423, 518 tail to 750 **solid**

a Diffuse reflectance.

**Figure 5:**
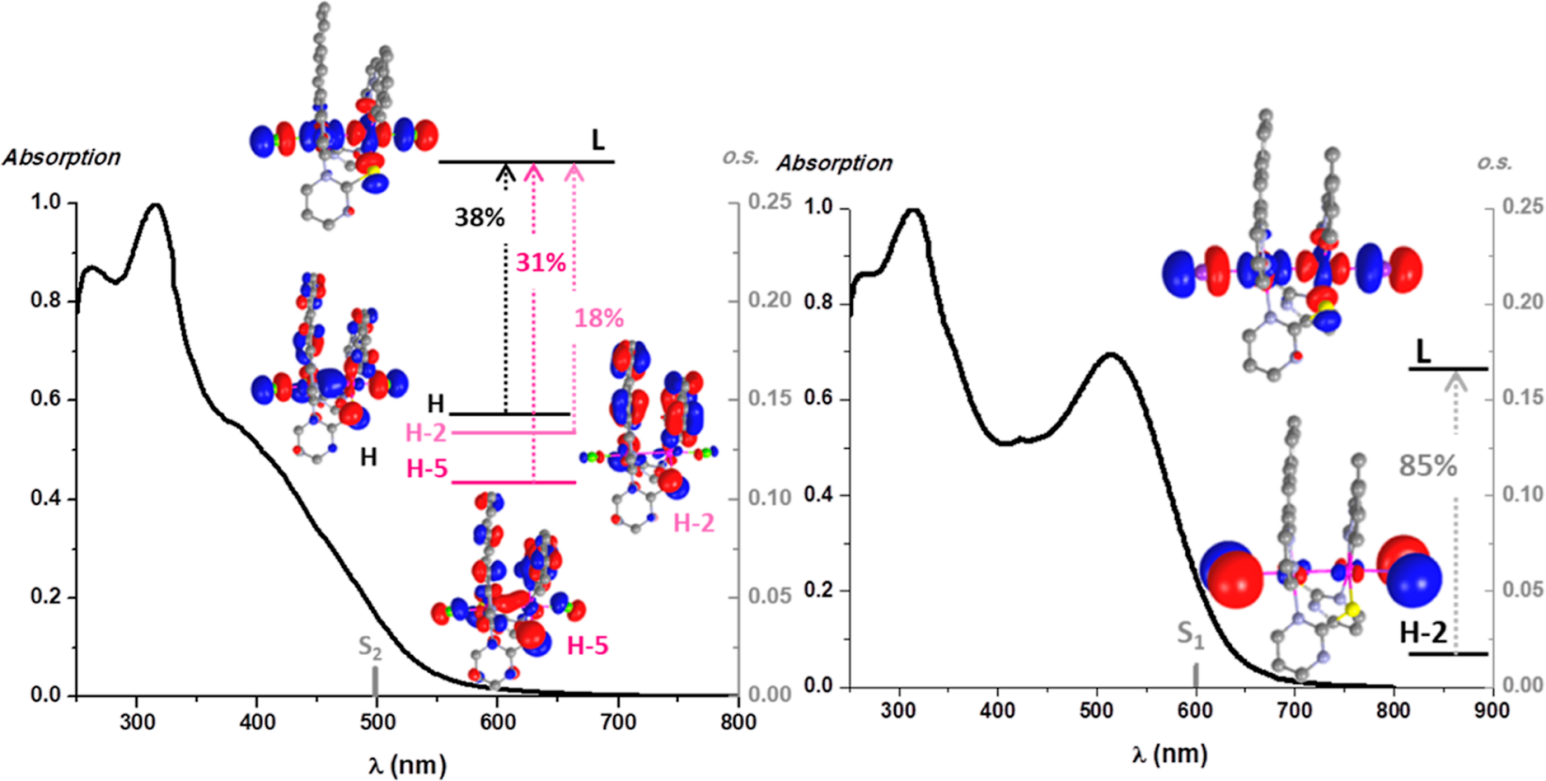
Normalized absorption spectra in the solid state, calculated transitions in the gas phase (gray bars), and molecular orbital plots (isoval. 0.03) for compounds **1-Cl** (left) and **1-I** (right).

To help with the UV–vis assignments, DFT and TD-DFT calculations were performed on **1**, **2**, **1-Cl**, and **1-I** (see Tables S2, S3 and Figures 4, 5, S15). As shown in Figures 4 right and S15, the selected calculated absorptions (S_1_ or S_2_) fit rather well with the experimental ones. In both Pt_2_(II,II) complexes, **1** and **2**, the main contribution to the S_1_ is the HOMO → LUMO transition, where the dσ* Pt–Pt orbital largely participates on the HOMO (∼75%) while the LUMO is completely centered on the S^∧^N bridging ligand (∼95%), see Tables S2 and S3. Thus, the lowest energy absorption for both, **1** and **2**, is attributed to a ^1^MML′CT [dσ*(Pt–Pt) → π*(S^∧^N)] excited state. This assignment is consistent with the red shift observed in the absorption bands in **2** with respect to **1**. The electron-withdrawing group CF_3_ in the S^∧^N lowers the LUMO level, decreasing the energy bandgap.

When comparing the lowest energy absorptions of **1** and **2** (λ_abs_: 405 and 404 nm for **1** and **2**), in solution of CH_2_Cl_2_, with those of the analogous bzq derivatives, [{Pt(bzq)(μ-S^∧^N^R^)}_2_] (λ_abs_: 496 nm R = H^39^ and 486 nm R = CF_3_^43^), a clear shift to higher energies is observed. As indicated before, we have carried out DFT calculations on complex [{Pt(bzq)(μ-S^∧^N^R^)}_2_] (R = H, 2-mercaptopyrimidine)^39^ and compared them with those of complex **1** (Figure S13). They showed that the HOMO in both complexes is constructed from dσ* orbitals but that of complex [{Pt(bzq)(μ-S^∧^N)}_2_] lays at a higher energy (−4.99 eV) than the one in **1** (−5.10 eV), indicating a better overlapping of the d_*z*^2^_ Pt orbitals in the former. However, the LUMO in complex [{Pt(bzq)(μ-S^∧^N)}_2_] (−1.585 eV) is mostly located on the bzq ligand, whereas in complex **1** (−1.25 eV), it is based on the S^∧^N bridging ligand. These calculations confirmed that the greater aromatic system of the benzoquinolinate ligand with respect to the naphthyl pyrazolate sharply changes the nature of the LUMO and leads to major stabilization. Also, this allows a greater π-backdonation from the Pt center to the bzq system, provoking a shortening of the Pt···Pt distance. Both effects lead to a decrease of the HOMO–LUMO energy gap and consequently, a red shift of lowest energy absorption along with a change in its nature, ^1^MML′CT [dσ*(Pt–Pt) → π*(S^∧^N)] in **1** and **2**, but ^1^MMLCT[dσ*(Pt–Pt) → π*(C^∧^N)] in the bzq derivatives. All this indicates the great influence of the C∧N ligand in the optical properties of half-lantern complexes.

In the Pt_2_(III,III) complexes, we will focus on the S_1_ transition [H-2 → LUMO (85%)] for **1-I** and on the S_2_ for **1-Cl**, in view of the negligible oscillator strength (o.s.) of S_1_, in this case. So, by analyzing the transitions involved in S_2_, we can observe certain orbital mixing for **1-Cl**: HOMO → LUMO (38%), H-5 → LUMO (31%) and H-2 → LUMO (18%). As illustrated in Figure 5 and according to the frontier orbital compositions (Table S2), the lowest energy absorption would be mainly attributed to mixed ^1^LL′MMCT[π(C^∧^N/S^∧^N) → dσ*(Pt–Pt)]/^1^LL′XT[π(C^∧^N/S^∧^N) → π*(X)] for **1-Cl** but for **1-I** to a ^1^XMMCT[π(X) → dσ*(Pt–Pt)] transition.

#### Emission Spectra

The photoluminescent properties were examined under an argon atmosphere in the solid state (Table 4). Unlike analogous compounds [{Pt(bzq)(μ-S^∧^N^R^)}_2_] (R = H,^39^ CF_3_^43^), complex **1** shows at 298 K an emission at *ca* 600 nm (Φ_PL_: 1.6%; τ: 0.1 μs) that becomes more structured and exhibits longer relaxation time when cooling down to 77 K (τ: 1.9 μs, see Figure S16). According to the decay times and the calculated spin density distribution for the T_1_ state (see Figure S16 and Tables 4 and S4), the phosphorescent emission of **1** is attributed to a ^3^MML′CT [dσ*(Pt–Pt) → π*(S^∧^N)] excited state. This is coherent with the shortening of the Pt–Pt distance from 2.964 Å in S_0_ to 2.820 Å in T_1_ due to the electron promotion from an antibonding orbital (dσ*Pt_2_) upon excitation (Table S5). However, no emission was detected for complex **2** either at room or low temperatures. Taking into account the DFT calculations and the application of the Energy Gap Law to Pt(II) chromophores,^62^ the electron-withdrawing CF_3_ group in the S^∧^N ligands decreases the energy bandgap in **2**, which can easily lead thermal deactivation processes. Besides, since the calculated spin density distribution for the T_1_ state is located mostly on the S∧N ligand (Table S4), the CF_3_ substituent can provide more vibrational and rotational motions, increasing nonradiative pathways.

**Table 4:** Photophysical Data in the Solid State

comp	*T* [K]	λ_ex_ [nm]	λ_em_ [nm] (τ [μs])
**1**	298	455	595 (0.1)
	77	455	572_max_, 614 (1.9)
**1-Cl**	298	500	985 (0.023)
	77	450	945 (3.222)
**1-Br**	298	500	985 (0.033)
	77	450	950 (3.042)
**1-I**	298	525	1070 (0.014)
	77	450	1055 (0.865)
**2-I**	298	—	—
	77	450	1050 (0.635)

**Figure 6:**
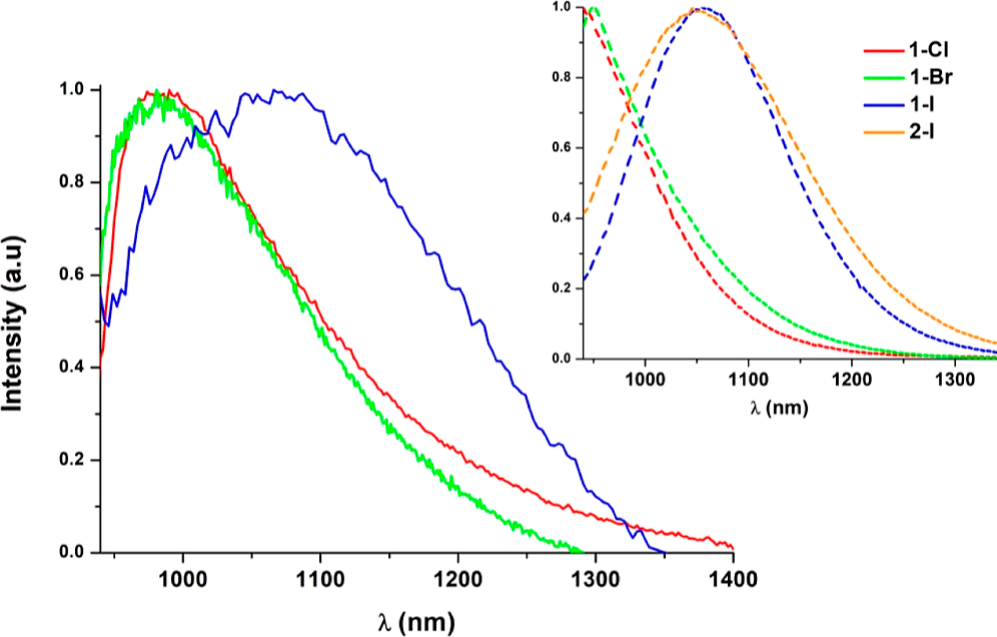
Normalized emission spectra in the solid state at 298 K (—). Inset: normalized emission spectra at 77 K (---).

Strikingly, powdery samples of the Pt_2_(III,III) compounds (**1-Cl**, **1-Br**, **1-I**) show at room temperature NIR emissions with maxima ranging from 985 to 1070 nm that are strongly dependent on the axial ligand X (Figure 6). Upon cooling down to 77 K, these emissions experience a hypsochromic shift and undergo a 100-fold increase in their lifetime decays (Table 4). A similar increment was also observed in related complexes [Pt_2_(HPO_4_)_4_X_2_]^4–^ (X = Cl, Br).^45^ Compound **2-I** shows no emission in the solid state at room temperature but it does at 77 K with λ_max_ and τ analogous to those of **1-I** (see Figure 6, inset). As observed in related iodo-complexes of Pt_2_(III)^47^ and in other Pt(IV)^63,64^ compounds, these exhibit weak emissions or even no emissions at all due to the effect of the iodo ligand. By analyzing the population of the frontier orbitals (Table S2), we observed that the higher π-donation of the *I* implies a greater participation along with a minor contribution of the metal center with respect to the chloro-derivatives. Therefore, the spin–orbit coupling induced by the Pt heavy atom would be less effective in the iodo-complexes, leading to less emissive compounds. In addition, the CF_3_ group may increase the nonradiative pathways in complex **2-I** with regard to **1-I**.

As stated before, the Pt_2_(III,III) complexes have been typically reported as nonemissive with the exception of very few examples that emit in the red^38,44,45^ and NIR^47^ spectral regions. If we compare them with our dinuclear Pt(III) systems, we clearly observe that our emissions are pushed further in the NIR. See as an example the emission bands in the solid state at 298 K for **1-Cl** and **1-I** (985 and 1070 nm, respectively) compared to those of [Pt_2_(μ-As^∧^C)_4_X_2_] (X = Cl, 915 nm and X = I, nonemissive at rt).^47^

With the aim of gathering more information about the origin of these emissions, we calculated the spin-density distributions in the optimized first excited states (T_1_) (Figure S17 and Table S4). They are located along the X–Pt–Pt-X axis, essentially on the X ligand (0.778 **1-Cl**, 1.031 **1-I**) and the Pt center (0.857 **1-Cl**, 0.669 **1-I**). There are also minor contributions from the N^∧^S (0.237 **1-Cl**, 0.184 **1-I**) and C^∧^N (0.128 **1-Cl**, 0.116 **1-I**) groups. Thus, the emissions would be mainly attributed to ^3^XMMCT [σ(X) → dσ*(Pt–Pt)] excited states with a minor contribution of the S^∧^N^R^ groups that become slight more important in the chloro derivatives. This assignment would be in close agreement with those reported for [Pt_2_(μ-As^∧^C)_4_X_2_] (X = Cl, Br, I), which were associated with an increasing admixture of XMMCT [σ(X) → dσ*(Pt–Pt)] with MC [dσ → dσ*] going from Cl-/Br-to the I-derivatives.^47^ Also for further confirmation, we analyzed the optimized geometries of the T_1_ states for **1-Cl** and **1-I**. They show a similar structure to that of the S_0_ state but with a substantial elongation of the Pt–Pt distance from 2.675 Å (S_0_) to 2.988 Å (T_1_) for **1-Cl** as an example (see Table S5 for **1-I**). This would be consistent with a decrease in the Pt–Pt BO as a consequence of the electron promoted to the dσ*(Pt)_2_ orbital in the excitation process. To evaluate this Pt–Pt bonding, Mayer BO analyses were carried out in the S_0_ and T_1_ states. As listed in Table S5, the BO of **1-Cl** in the S_0_ is 0.61, whereas in T_1_, it is 0.27. Then, this Pt–Pt interaction is significantly weakened in the first excited state of both complexes, **1-Cl** and **1-I**, which would be supporting the ^3^XMMCT character of these emissions. Finally, if we compare the calculated spin density on the Pt center in complexes **1-I** (0.669) and **1-Cl** (0.857), a weaker spin–orbit effect induced by the Pt heavy atom would be expected for **1-I**, which would decrease the kr. This is in accordance with our experimental results for the iodo-complexes **1-I** and **2-I**.

## Conclusions

Half-lantern dinuclear Pt(II) complexes bearing naphthyl-pyrazole as the cyclometalated ligand have been prepared and oxidized with haloforms to give the corresponding diplatinum(III) derivatives with halogenides as axial capping ligands. The Pt_2_(II,II) complex **1** shows a yellow emission at both RT and 77 K, while the oxidized Pt_2_(III,III) counterparts display emission bands around 1000 nm that strongly depend on the nature of the axial ligand. The C^∧^N-skeleton seems to be responsible for the encountered differences with respect to analogous half-lantern complexes with bzq as the C^∧^N cyclometalated group. First is the reactivity of Pt_2_(II,II) compounds toward haloforms. In this case, light is essential to initiate and complete the reaction of **1** with CHBr_3_. Second, it affects the nature of the absorption and emission bands of Pt_2_(II,II) compounds that are MML′CT [dσ*(Pt–Pt) → π*(S^∧^N)] based. In the third place and finally, we have obtained Pt_2_(III,III) compounds that exhibit long-wavelength NIR bands (∼1000 nm) at room temperature that are mainly due to ^3^XMMCT [σ(X) → dσ*(Pt–Pt)] excited states. These discrete molecules with a semirigid dinuclear structure held together by two bridging groups and a metal–metal bond constitute an approachable platform to achieve emitters in the NIR-II spectral region. By regulating the nature of the axial ligands as well as the cyclometalating and bridging groups, we can introduce structural modifications that certainly affect the photophysical properties. These results contribute significantly to opening out the possibilities for the highly desired NIR-II emitters.

## Supporting Information Available

Experimental appendix including photophysical and computational methods and X-ray crystallography details (2299209–2299212); NMR spectra and X-ray diffraction figures for structural characterization; theoretical calculations; and photophysical properties (PDF)
Cartesian coordinates of the DFT-optimized structures for complexes **1**, **2**, **1-Cl**, **1-I**, and [{Pt(bzq)(μ-S∧N)_2_}] (HS∧*N* = 2-mercaptopyrimidine) (XYZ)

## Supplementary Material

ic3c04314_si_001.pdf

ic3c04314_si_002.xyz

## References

[ref1] Wang S.-F.; Su B.-K.; Wang X.-Q.; Wei Y.-C.; Kuo K.-H.; Wang C.-H.; Liu S.-H.; Liao L.-S.; Hung W.-Y.; Fu L.-W.; Chuang W.-T.; Qin M.; Lu X.; You C.; Chi Y.; Chou P.-T. Polyatomic Molecules with Emission Quantum Yields > 20% Enable Efficient Organic Light-Emitting Diodes in the NIR(II) Window. Nat. Photonics 2022, 16, 843–850. 10.1038/s41566-022-01079-8.

[ref2] Foucault-Collet A.; Gogick K. A.; White K. A.; Villette S.; Pallier A.; Collet G.; Kieda C.; Li T.; Geib S. J.; Rosi N. L.; Petoud S. Lanthanide Near Infrared Imaging in Living Cells With Yb^3+^ Nano Metal Organic Frameworks. Proc. Natl. Acad. Sci. U.S.A. 2013, 110, 17199–17204. 10.1073/pnas.1305910110. 24108356 PMC3808657

[ref3] Martinić I.; Eliseeva S. V.; Nguyen T. N.; Pecoraro V. L.; Petoud S. Near-Infrared Optical Imaging of Necrotic Cells by Photostable Lanthanide-Based Metallacrowns. J. Am. Chem. Soc. 2017, 139, 8388–8391. 10.1021/jacs.7b01587. 28613848

[ref4] Bai G.; Yang Z.; Lin H.; Jie W.; Hao J. Lanthanide Yb/Er co-doped Semiconductor Layered WSe_2_ Nanosheets with Near-infrared Luminescence at Telecommunication Wavelengths. Nanoscale 2018, 10, 9261–9267. 10.1039/C8NR01139G. 29736531

[ref5] Smith A. M.; Mancini M. C.; Nie S. Second Window for in Vivo Imaging. Nat. Nanotechnol. 2009, 4, 710–711. 10.1038/nnano.2009.326. 19898521 PMC2862008

[ref6] Monro S.; Colon K. L.; Yin H.; Roque J. III; Konda P.; Gujar S.; Thummel R. P.; Lilge L.; Cameron C. G.; McFarland S. A. Transition Metal Complexes and Photodynamic Therapy from a Tumor-Centered Approach: Challenges, Opportunities, and Highlights from the Development of TLD1433. Chem. Rev. 2019, 119, 797–828. 10.1021/acs.chemrev.8b00211. 30295467 PMC6453754

[ref7] Zhang K. Y.; Yu Q.; Wei H.; Liu S.; Zhao Q.; Huang W. Long-Lived Emissive Probes for Time-Resolved Photoluminescence Bioimaging and Biosensing. Chem. Rev. 2018, 118, 1770–1839. 10.1021/acs.chemrev.7b00425. 29393632

[ref8] Zhen X.; Qu R.; Chen W.; Wu W.; Jiang X. The Development of Phosphorescent Probes for *in vitro* and *in vivo* Bioimaging. Biomater. Sci. 2021, 9, 285–300. 10.1039/D0BM00819B. 32756681

[ref9] Yam V. W. W.; Au V.; Leung S. Light-Emitting Self-Assembled Materials Based on d^8^ and d^10^ Transition Metal Complexes. Chem. Rev. 2015, 115, 7589–7728. 10.1021/acs.chemrev.5b00074. 26158432

[ref10] Kang J.; Zhang X. H.; Zhou H.; Gai X.; Jia T.; Xu L.; Zhang J.; Li Y.; Ni J. 1-D ″Platinum Wire″ Stacking Structure Built of Platinum(II) Diimine Bis(σ-acetylide) Units with Luminescence in the NIR Region. Inorg. Chem. 2016, 55, 10208–10217. 10.1021/acs.inorgchem.6b01426. 27681832

[ref11] Su M.; Liu S.; Zhang J.; Meng C.; Ni J. The triple-stimuli-responsive luminescence switching properties and application of a square-planar platinum(II) complex. Dyes Pigm. 2022, 200, 110139 10.1016/j.dyepig.2022.110139.

[ref12] Su M.; Kang J.; Liu S.; Meng C.; Li Y.; Zhang J.; Ni J. Strategy for Achieving Long-Wavelength Near-Infrared Luminescence of Diimineplatinum(II) Complexes. Inorg. Chem. 2021, 60, 3773–3780. 10.1021/acs.inorgchem.0c03529. 33615779

[ref13] Wei Y.-C.; Kuo K.-H.; Chi Y.; Chou P.-T. Efficient Near-Infrared Luminescence of Self-Assembled Platinum(II) Complexes: From Fundamentals to Applications. Acc. Chem. Res. 2023, 56, 689–699. 10.1021/acs.accounts.2c00827. 36882976

[ref14] Zhang X.; Chi Z.; Zhang Y.; Liu S.; Xu J. Recent Advances in Mechanochromic Luminescent Metal Complexes. J. Mater. Chem. C 2013, 1, 3376–3390. 10.1039/c3tc30316k.

[ref15] Connick W. B.; Henling L. M.; Marsh R. E.; Gray H. B. Emission Spectroscopic Properties of the Red Form of Dichloro(2,2′-bipyridine)platinum(II). Role of Intermolecular Stacking Interactions. Inorg. Chem. 1996, 35, 6261–6265. 10.1021/ic960511f.

[ref16] Wenger O. S. Vapochromism in Organometallic and Coordination Complexes: Chemical Sensors for Volatile Organic Compounds. Chem. Rev. 2013, 113, 3686–3733. 10.1021/cr300396p. 23317416

[ref17] Yoshida M.; Kato M. Regulation of Metal-Metal Interactions and Chromic Phenomena of Multi-Decker Platinum Complexes Having π-Systems. Coord. Chem. Rev. 2018, 355, 101–115. 10.1016/j.ccr.2017.07.016.

[ref18] Koshiyama T.; Omura A.; Kato M. Redox-controlled Luminescence of a Cyclometalated Dinuclear Platinum Complex Bridged with Pyridine-2-thiolate Ions. Chem. Lett. 2004, 33, 1386–1387. 10.1246/cl.2004.1386.

[ref19] Saito K.; Hamada Y.; Takahashi H.; Koshiyama T.; Kato M. Organic Light-Emitting Diodes Based on a Binuclear Platinum(II) Complex. Jpn. J. Appl. Phys. 2005, 44, L500 10.1143/JJAP.44.L500.

[ref20] Wang Z.; Jiang L.; Liu Z.-P.; Gan C. R. R.; Liu Z. L.; Zhang X. H.; Zhao J.; Hor T. S. A. Facile Formation and Redox of Benzoxazole-2-Thiolate-Bridged Dinuclear Pt(II/III) Complexes. Dalton Trans. 2012, 41, 12568 10.1039/c2dt31070h. 22960665

[ref21] Roy S.; Lopez A. A.; Yarnell J. E.; Castellano F. N. Metal-Metal-to-Ligand Charge Transfer in Pt(II) Dimers Bridged by Pyridyl and Quinoline Thiols. Inorg. Chem. 2022, 61, 121–130. 10.1021/acs.inorgchem.1c02469. 34955020

[ref22] Katlenok E. A.; Zolotarev A. A.; Balashev K. P. Binuclear Complexes of Pt(II) with Platinated 2-Phenylbenzothiazole and Bridged Derivatives of Pyridin- and Benzothiazol-2-Thiols. Russ. J. Gen. Chem. 2014, 84, 1593–1598. 10.1134/S107036321408026X.

[ref23] Zhu Y.; Luo K.; Zhao L.; Ni H.; Li Q. Binuclear Platinum(II) Complexes Based on 2-Mercaptobenzothiazole 2-Mercaptobenzimidazole and 2-Hydroxpyridine as Brigding Ligands: Red and Near-Infrared Luminescence Originated from MMLCT Transition. Dyes Pigm. 2017, 145, 144–151. 10.1016/j.dyepig.2017.05.052.

[ref24] Su N.; Meng F.; Wang P.; Liu X.; Zhu M.; Zhu W.; Su S.; Yu J. Near-infrared Emission from Binuclear Platinum (II) Complexes Containing Pyrenylpyridine and Pyridylthiolate Units: Synthesis, Photo-Physical and Electroluminescent Properties. Dyes Pigm. 2017, 138, 162–168. 10.1016/j.dyepig.2016.11.037.

[ref25] Wu X.; Liu Y.; Wang Y.; Wang L.; Tan H.; Zhu M.; Zhu W.; Cao Y. Highly Efficient Near-Infrared Emission from Binuclear Cyclo-Metalated Platinum Complexes Bridged with 5-(4-Octyloxyphenyl)-1,3,4-oxadiazole-2-thiol in Pleds. Org. Electron. 2012, 13, 932–937. 10.1016/j.orgel.2012.02.004.

[ref26] Xiong W.; Meng F.; Tan H.; Wang Y.; Wang P.; Zhang Y.; Tao Q.; Su S.; Zhu W. Dinuclear Platinum Complexes Containing Aryl-Isoquinoline and Oxadiazole-thiol with an Efficiency of Over 8.8%: In-Depth Investigation of the Relationship Between Their Molecular Structure and Near-Infrared Electroluminescent Properties in Pleds. J. Mater. Chem. C 2016, 4, 6007–6015. 10.1039/C6TC00825A.

[ref27] Xiong W.; Meng F.; You C.; Wang P.; Yu J.; Wu X.; Pei Y.; Zhu W.; Wang Y.; Su S. Molecular Isomeric Engineering of Naphthyl-Quinoline-Containing Dinuclear Platinum Complexes to Tune Emission from Deep Red to Near Infrared. J. Mater. Chem. C 2019, 7, 630–638. 10.1039/C8TC05263H.

[ref28] Chaaban M.; Chi Y.-C.; Worku M.; Zhou C.; Lin H.; Lee S.; Ben-Akacha A.; Lin X.; Huang C.; Ma B. Thiazol-2-thiolate-Bridged Binuclear Platinum(II) Complexes with High Photoluminescence Quantum Efficiencies of up to Near Unity. Inorg. Chem. 2020, 59, 13109–13116. 10.1021/acs.inorgchem.0c01256. 32865987

[ref29] Shahsavari H. R.; Lalinde E.; Moreno M. T.; Niazi M.; Kazemi S. H.; Abedanzadeh S.; Barazandeh M.; Halvagar M. R. Half-lantern Cyclometalated Pt(II) and Pt(III) Complexes with Bridging Heterocyclic Thiolate Ligands: Synthesis, Structural Characterization, and Electrochemical and Photophysical Properties. New J. Chem. 2019, 43, 7716–7724. 10.1039/C9NJ00969H.

[ref30] Yamada Y.; Matsumoto R.; Kori D.; Koikawa M. Syntheses, Crystal Structures, and Solid-State Spectroscopic Properties of Dinuclear Cyclometallated Platinum(II) Complexes with Mercaptobenzoazoles as Bridging Ligands. Inorg. Chim. Acta 2021, 515, 120049 10.1016/j.ica.2020.120049.

[ref31] Katlenok E. A.; Kryukov D. M.; Kurtsevich A. E.; Degtyarenko K. M.; Valiev R. R.; Levin O. V.; Kukushkin V. Y.; Rozhkov A. V. Incorporation of a Fluorine Atom in a Bridging Ligand of Half-Lantern Pt^II^_2_ Complexes Provides up to 10-Fold Enhancement of Electroluminescence Brightness. Inorg. Chem. 2023, 62, 11080–11094. 10.1021/acs.inorgchem.3c01136. 37382990

[ref32] Chakraborty A.; Yarnell J. E.; Sommer R. D.; Roy S.; Castellano F. N. Excited-State Processes of Cyclometalated Platinum(II) Charge-Transfer Dimers Bridged by Hydroxypyridines. Inorg. Chem. 2018, 57, 1298–1310. 10.1021/acs.inorgchem.7b02736. 29336558

[ref33] Zhu Y.; Luo K.; Li X.; Wang H.; Yang C.; Ni H.; Li Q. Four New Binuclear Platinum (II) Complexes with 2-(1H)-Quinolinone as Bridging Ligands: Synthesis, Crystal Structure and Photophysical Properties. J. Lumin. 2018, 204, 296–302. 10.1016/j.jlumin.2018.08.010.

[ref34] Wang S. F.; Fu L.-W.; Wei Y.-C.; Liu S.; Lin J.; Lee G.; Chou P.-T.; Huang J.; Wu C.-I.; Yuan Y.; Lee C.-S.; Chi Y. Near-Infrared Emission Induced by Shortened Pt-Pt Contact: Diplatinum(II) Complexes with Pyridyl Pyrimidinato Cyclometalates. Inorg. Chem. 2019, 58, 13892–13901. 10.1021/acs.inorgchem.9b01754. 31565936

[ref35] Zhang Y. M.; Miao J. S.; Xiong J. F.; Li K.; Yang C. L. Rigid Bridge-Confined Double-Decker Platinum(II) Complexes Towards High-Performance Red and Near-Infrared Electroluminescence. Angew. Chem., Int. Ed. 2022, 61, e202113718 10.1002/anie.202113718. 34734464

[ref36] Wen Z.; Xu Y.; Song X.-.; Miao J. S.; Zhang Y.; Li K.; Yang C. L. Approaching the Shortest Intermetallic Distance of Half-Lantern Diplatinum(II) Complexes for Efficient and Stable Deep-Red Organic Light-Emitting Diodes. Adv. Opt. Mater. 2023, 11, 2300201 10.1002/adom.202300201.

[ref37] Park H. J.; Boelke C. L.; Cheong P. H.-Y.; Hwang D.-H. Dinuclear Pt(II) Complexes with Red and NIR Emission Governed by Ligand Control of the Intramolecular Pt-Pt Distance. Inorg. Chem. 2022, 61, 5178–5183. 10.1021/acs.inorgchem.1c03967. 35320671

[ref38] Roundhill D. M.; Gray H. B.; Che C. M. Pyrophosphito-Bridged Diplatinum Chemistry. Acc. Chem. Res. 1989, 22, 55–61. 10.1021/ar00158a002.

[ref39] Sicilia V.; Baya M.; Borja P.; Martín A. Oxidation of Half-Lantern Pt_2_(II,II) Compounds by Halocarbons. Evidence of Dioxygen Insertion into a Pt(III)-CH_3_ Bond. Inorg. Chem. 2015, 54, 7316–7324. 10.1021/acs.inorgchem.5b00846. 26197039

[ref40] Bellitto C.; Flamini A.; Gastaldi L.; Scaramuzza L. Halogen Oxidation of Tetrakis(Dithioacetato)Diplatinum(II) Complexes, Pt_2_(CH_3_CS_2_)_4_-Synthesis and Characterization of Pt_2_(CH_3_CS_2_)_4_X_2_ (X = Cl, Br, I) and Structural, Electrical, and Optical-Properties of Linear-Chain (M-I)Tetrakis(Dithioacetato)Diplatinum, Pt_2_(CH_3_CS_2_)_4_I. Inorg. Chem. 1983, 22, 444–449. 10.1021/ic00145a015.

[ref41] Sicilia V.; Forniés J.; Casas J. M.; Martin A.; López J. A.; Larraz C.; Borja P.; Ovejero C.; Tordera D.; Bolink H. Highly Luminescent Half-Lantern Cyclometalated Platinum(II) Complex: Synthesis, Structure, Luminescence Studies, and Reactivity. Inorg. Chem. 2012, 51, 3427–3435. 10.1021/ic201910t. 22360773

[ref42] Sicilia V.; Borja P.; Casas J. M.; Fuertes S.; Martin A. Selective Synthesis of new Half-lantern Benzoquinolate Platinum Complexes. DFT and Photophysical Studies on the Platinum (II,II) derivative. J. Organomet. Chem. 2013, 731, 10–17. 10.1016/j.jorganchem.2013.01.027.

[ref43] Sicilia V.; Borja P.; Martín A. Half-Lantern Pt(II) and Pt(III) Complexes. New Cyclometalated Platinum Derivatives. Inorganics 2014, 2, 508–523. 10.3390/inorganics2030508.

[ref44] Wu X.; Chen D.-G.; Liu D.; Liu S.-H.; Shen S.-W.; Wu C.-I.; Xie G.; Zhou J.; Huang Z.-X.; Huang C.-Y.; Su S.-J.; Zhu W.; Chou P.-T. Highly Emissive Dinuclear Platinum(III) Complexes. J. Am. Chem. Soc. 2020, 142, 7469–7479. 10.1021/jacs.9b13956. 32223139

[ref45] Shin Y. G. K.; Miskowski V. M.; Nocera D. G. Luminescence from diplatinum(III) tetraphosphate complexes: dynamics of emissive dσ* excited states. Inorg. Chem. 1990, 29, 2308–2313. 10.1021/ic00337a024.

[ref46] Stiegman A. E.; Miskowski V. M.; Gray H. B. Metal-Metal Excited-State Emission from Binuclear Platinum(III) Complexes. J. Am. Chem. Soc. 1986, 108, 2781–2782. 10.1021/ja00270a062. 22283300

[ref47] Bennett M. A.; Bhargava S. K.; Cheng E. C.-C.; Lam W. H.; Lee T. K.-M.; Priver S. H.; Wagler J.; Willis A. C.; Yam V. W.-W. Unprecedented Near-Infrared (NIR) Emission in Diplatinum(III) (d(7)-d(7)) Complexes at Room Temperature. J. Am. Chem. Soc. 2010, 132, 7094–7103. 10.1021/ja1002313. 20433140

[ref48] Paziresh S.; Sicilia V.; Ara I.; Martin A.; Fuertes S. The Influence of Cyclometalated Ligand Motifs on the Solid-State Assemblies and Luminescent Properties of Pt(II)-Tl(I) Complexes. Organometallics 2019, 38, 3804–3815. 10.1021/acs.organomet.9b00500.

[ref49] Zhao Y.; Truhlar D. G. The M06 Suite Of Density Functionals For Main Group Thermochemistry, Thermochemical Kinetics, Noncovalent Interactions, Excited States, And Transition Elements: Two New Functionals And Systematic Testing Of Four M06-Class Functionals And 12 Other Functionals. Theor. Chem. Acc. 2008, 120, 215–241. 10.1007/s00214-007-0310-x.

[ref50] Grimme S.; Antony J.; Ehrlich S.; Krieg H. A Consistent And Accurate Ab Initio Parametrization Of Density Functional Dispersion Correction (DFT-D) For The 94 Elements H-Pu. J. Chem. Phys. 2010, 132, 154104 10.1063/1.3382344. 20423165

[ref51] Andrae D.; Häußermann U.; Dolg M.; Stoll H.; Preuß H. Energy-Adjustedab Initio Pseudopotentials For The Second And Third Row Transition Elements. Theor. Chim. Acta 1990, 77, 123 10.1007/BF01114537.

[ref52] Ditchfield R.; Hehre W. J.; Pople J. A. Self-Consistent Molecular-Orbital Methods. IX. An Extended Gaussian-Type Basis for Molecular-Orbital Studies of Organic Molecules. J. Chem. Phys. 1971, 54, 724–728. 10.1063/1.1674902.

[ref53] Hariharan P. C.; Pople J. A. The Influence Of Polarization Functions On Molecular Orbital Hydrogenation Energies. Theor. Chim. Acta 1973, 28, 213–222. 10.1007/BF00533485.

[ref54] Frisch M. J.; Gaussian 09; Gaussian, Inc.: Wallingford, CT, USA, 2013.

[ref55] Aoki R.; Kobayashi A.; Chang H.-C.; Kato M. Structures and Luminescence Properties of Cyclometalated Dinuclear Platinum(II) Complexes Bridged by Pyridinethiolate Ions. Bull. Chem. Soc. Jpn. 2011, 84, 218–225. 10.1246/bcsj.20100304.

[ref56] Ma B.; Djurovich P. I.; Garon S.; Alleyne B.; Thompson M. E. Platinum Binuclear Complexes as Phosphorescent Dopants for Monochromatic and White Organic Light-Emitting Diodes. Adv. Funct. Mater. 2006, 16, 2438–2446. 10.1002/adfm.200600614.

[ref57] Umakoshi K.; Kinoshita I.; Ichimura A.; Ooi S. Binuclear Platinum(II) and -(III) Complexes of Pyridine-2-Thiol and its 4-Methyl Analog. Synthesis, Structure, And Electrochemistry. Inorg. Chem. 1987, 26, 3551–3556. 10.1021/ic00268a027.

[ref58] Arnal L.; Fuertes S.; Martín A.; Baya M.; Sicilia V. A Cyclometalated N-Heterocyclic Carbene: The Wings of the First Pt_2_(II,II) Butterfly Oxidized by CHI3. Chem.—Eur. J. 2018, 24, 18743–18748. 10.1002/chem.201804013. 30273446

[ref59] Sicilia V.; Arnal L.; Fuertes S.; Martin A.; Baya M. Metal-Metal Cooperation in the Oxidation of a Flapping Platinum Butterfly by Haloforms: Experimental and Theoretical Evidence. Inorg. Chem. 2020, 59, 12586–12594. 10.1021/acs.inorgchem.0c01701. 32815727

[ref60] Roundhill D. M.; Dickson M. K.; Atherton S. J. Thermal and Photochemical Addition of Alkyl and Aryl Halides to Tetrakis(Μ-Pyrophosphito) Diplatinum(II) Tetraanion. J. Organomet. Chem. 1987, 335, 413–422. 10.1016/S0022-328X(00)99415-4.

[ref61] Che C. M.; Butler L. G.; Grunthaner P. J.; Gray H. B. Chemistry and Spectroscopy of Binuclear Platinum Diphosphite Complexes. Inorg. Chem. 1985, 24, 4662–4665. 10.1021/ic00220a047.

[ref62] Cummings S. D.; Eisenberg R. Tuning the Excited-State Properties of Platinum(II) Diimine Dithiolate Complexes. J. Am. Chem. Soc. 1996, 118, 1949–1960. 10.1021/ja951345y.

[ref63] López-López J. C.; Bautista D.; González-Herrero P. Luminescent Halido(aryl) Pt(IV) Complexes Obtained via Oxidative Addition of Iodobenzene or Diaryliodonium Salts to bis-Cyclometalated Pt(II) Precursors. Dalton Trans. 2021, 50, 13294–13305. 10.1039/D1DT02349G. 34499066

[ref64] Juliá F.; García-Legaz M. D.; Bautista D.; González-Herrero P. Influence of Ancillary Ligands and Isomerism on the Luminescence of Bis-cyclometalated Platinum(IV) Complexes. Inorg. Chem. 2016, 55, 7647–7660. 10.1021/acs.inorgchem.6b01100. 27438708

