# Supplementary material for: NIR-II Emission from Cyclometalated Dinuclear Pt(III) Complexes
Source: Inorg Chem. 2024 Mar 8;63(12):5470–80. doi: 10.1021/acs.inorgchem.3c04314 (PMC10966738; doi:10.1021/acs.inorgchem.3c04314)
Supplement: Supplementary file 1 — ic3c04314_si_001.pdf [file ic3c04314_si_001.pdf]

Supporting Information

NIR-II Emission from Cyclometalated Dinuclear Pt(III) Complexes

Irene Melendo,[a] Sara Fuertes,[a] Antonio Martín,[a] and Violeta Sicilia*[b]*

a. *Instituto de Síntesis Química y Catálisis Homogénea (ISQCH), CSIC - Universidad de Zaragoza, Pedro Cerbuna 12, 50009, Zaragoza (Spain) E-mail: sfuertes@unizar.es*

b. *Departamento de Química Inorgánica, Escuela de Ingeniería y Arquitectura de Zaragoza, Instituto de Síntesis Química y Catálisis Homogénea (ISQCH), CSIC - Universidad de Zaragoza, Campus Río Ebro, Edificio Torres Quevedo, 50018, Zaragoza (Spain). E-mail: sicilia@unizar.es*

CONTENTS **Page**

1. Experimental appendix: S3

1.1. Photophysical Methods S3

1.2. X-ray Crystallography Details S3

2. Structural characterization and reactivity S6

3. Photophysical properties and theoretical calculations S18

4. References S22

1. EXPERIMENTAL

1.1. Photophysical measurements. Diffuse reflectance UV-vis (DRUV) spectra of the solid samples were recorded on a JASCO V-670 UV-vis spectrophotometer equipped with a Harrick Praying mantis diffuse reflectance accessory. Spectral grade $BaSO_4$ was used as a reference material. The photoluminescence experiments were carried out on solid samples placed in quartz tubes under argon atmosphere. Steady-state photoluminescence spectra of **1** were recorded on a Jobin-Yvon Horiba Fluorolog FL-3-11 Tau 3 spectrofluorimeter. Emission lifetimes were recorded with a Datastation HUB-B with a nanoLED controller and software DAS6. The nanoLEDs employed for lifetime measurements were of 455 nm. The lifetime data were fitted using the Jobin-Yvon software package and the Origin Pro 8 program. Quantum yields were measured using the Hamamatsu Absolute PL Quantum Yield Measurement System C11347-11 at room temperature. NIR steady-state photoluminescence spectra were recorded in a FluoTime 300 spectrofluorometer equipped with a NIR-PMT detector (Hamamatsu H10330C-75) using a 300W Xe lamp or a 450 nm Picosecond Laser Diode for excitation. Time-resolve measurements in the NIR spectral region were recorded with a 450 nm Picosecond Laser Diode.

1.2. X-ray Crystallography

Crystal data and other details of the structure analyses are summarized in Table S1. Single crystals of **2** and **1-I** were obtained by slow diffusion of diethylether into saturated solutions of acetone (**2**) or dichloromethane (**1-I**). Single crystals of **1-Cl** and **2-I** were prepared from slow diffusion of *n*-hexane into saturated dichloromethane solutions. Crystals were mounted at the end of quartz fibres. X-ray intensity data were collected on an Oxford Diffraction Xcalibur diffractometer using graphite monochromated MoKα radiation (0.71073 Å). The diffraction frames were integrated

and corrected from absorption by using the CrysAlis RED program.[1] The structures were solved by Patterson and Fourier methods and refined by full-matrix least squares on F^2 with SHELXL.[2] All non-hydrogen atoms were assigned anisotropic displacement parameters. The positions of the hydrogen atoms were constrained to idealised geometries and assigned isotropic displacement parameters equal to 1.2 or 1.5 times the *U*iso values of their respective parent atoms. Full-matrix least-squares refinement of the models against F2 converged to final residual indices given in Table S1. CCDC Nos. 2299209-2299212 contain the supplementary crystallographic data for **2**, **1-Cl**, **1-I** and **2-I**.

Table S1: Crystallographic data.

	**2 · C_3H_6O**	**1-Cl · 1.25 CH_2Cl_2**	**1-I · $O(CH_2CH_3)_2$**	**2-I · CH_2Cl_2**
Empirical formula	$C_{39}H_{28}F_6N_8OPt_2S_2$	$C_{35.50}H_{27}Cl_5N_8Pt_2S_2$	$C_{38}H_{34}I_2N_8OPt_2S_2$	$C_{37}H_{24}Cl_2F_6I_2N_8Pt_2S_2$
Formula weight	1192.99	1197.20	1326.83	1473.64
Crystal system	Triclinic	Monoclinic	Tetragonal	Monoclinic
Space group	P -1	C 2/c	P $4_1$2 2	P 2_1/c
a (Å)	11.9916(3)	13.6074(8)	10.77727(14)	13.72340(16)
b (Å)	12.3145(3)	25.9588(10)	10.77727(14)	25.0292(2)
c (Å)	14.4645(4)	12.0213(6)	34.1578(7)	12.67456(15)
α (º)	70.688(2)	90	90	90
β (º)	69.331(2)	111.495(6)	90	103.5423(12
γ (º)	75.947(2)	90	90	90
Volume (Å^3)/Z	1866.72(8) / 2	3951.0(4) / 4	3967.41(13) / 4	4232.48(8) / 4
ρ (Mg/m^3)	2.122	2.013	2.221	2.313
μ (Mo-Kα) (mm^{-1})	7.674	7.556	8.748	8.353
F(000)	1136	2276	2480	2736
Crystal size (mm^3)	0.20 x 0.18 x 0.09	0.190 x 0.040 x 0.030	0.220 x 0.190 x 0.180	0.360 x 0.290 x 0.200
Theta range (º)	2.66 to 28.40	2.851 to 26.499	2.602 to 30.405	2.628 to 29.511
Reflections collected	28960	15079	31039	111235
Independent reflections [R(int)]	8114 [0.0307]	4100 [0.0725]	5589 [0.00512]	11011 [0.0418]
Final R_1, wR_2 [I>2sigma(I)]^a	0.0246, 0.0507	0.0465, 0.0894	0.0335, 0.0680	0.0311, 0.0730
R_1, wR_2 (all data)^a	0.0305, 0.0533	0.0834, 0.1030	0.0418, 0.0714	0.0413, 0.0777
Absolute structure parameter	—	—	-0.010(3)	—
GOF (F^2)^b	1.034	1.022	1.059	1.044
Largest diff. peak and hole/ e.Å^{-3}	1.427 and -1.647	1.576 and -0.975	1.030 and -0.877	2.261 and -1.627

^{a} $R_1 = \sum(|F_o| - |F_c|) / \sum |F_o|$. $wR_2 = [\sum w (F_o^2 - F_c^2)^2 / \sum w(F_o^2)^2]^{1/2}$

^{b} Goodness-of-fit = $[\sum w (F_o^2 - F_c^2)^2 / (n_{obs} - n_{param})]^{1/2}$.

2. Structural characterization and reactivity

(a)

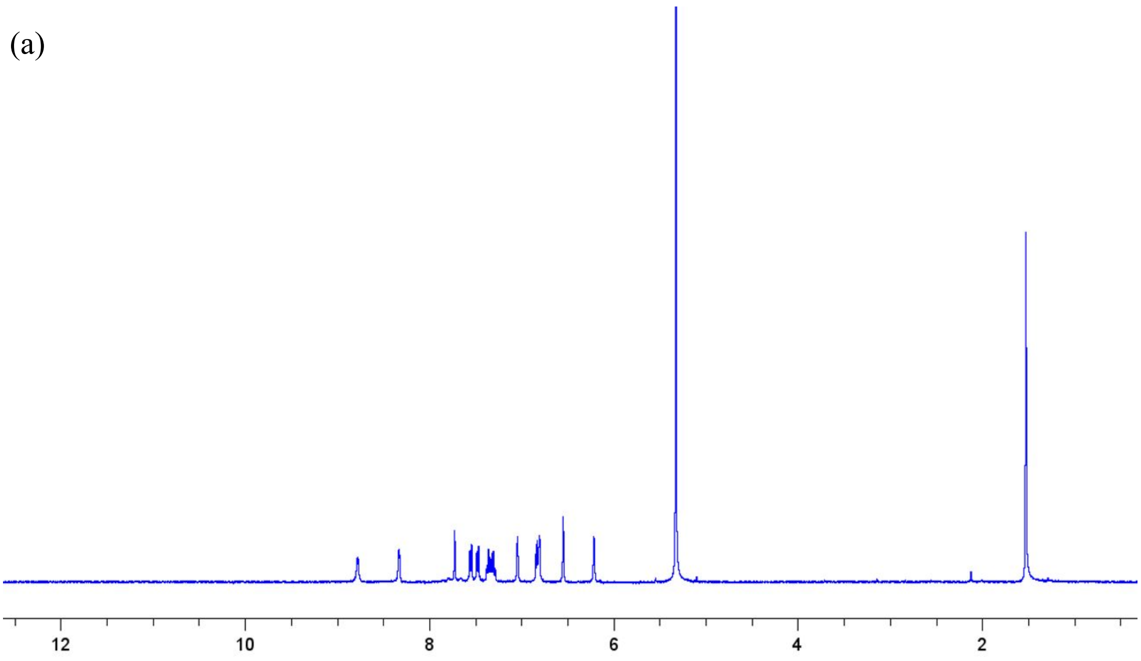

(b)

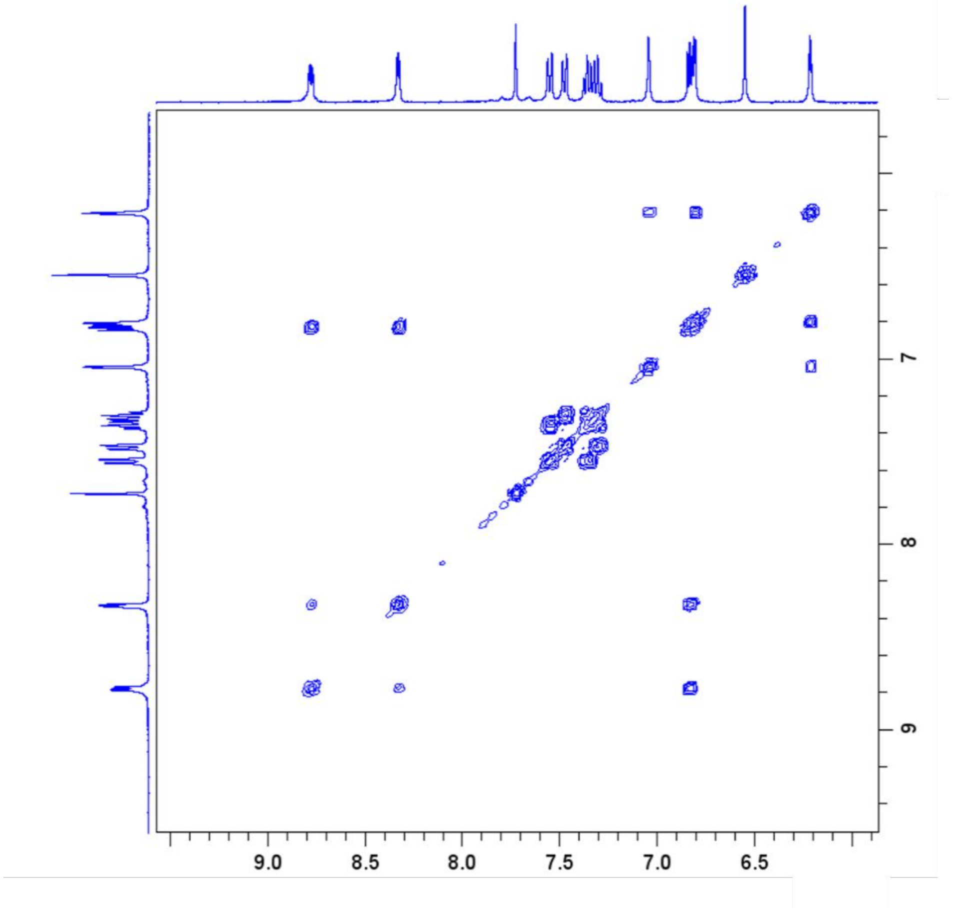

Figure S1. ^{1}H (a) and ^{1}H-^{1}H COSY (b) NMR spectra of **1** in CD_2Cl_2.

(a)

(b)

(c)

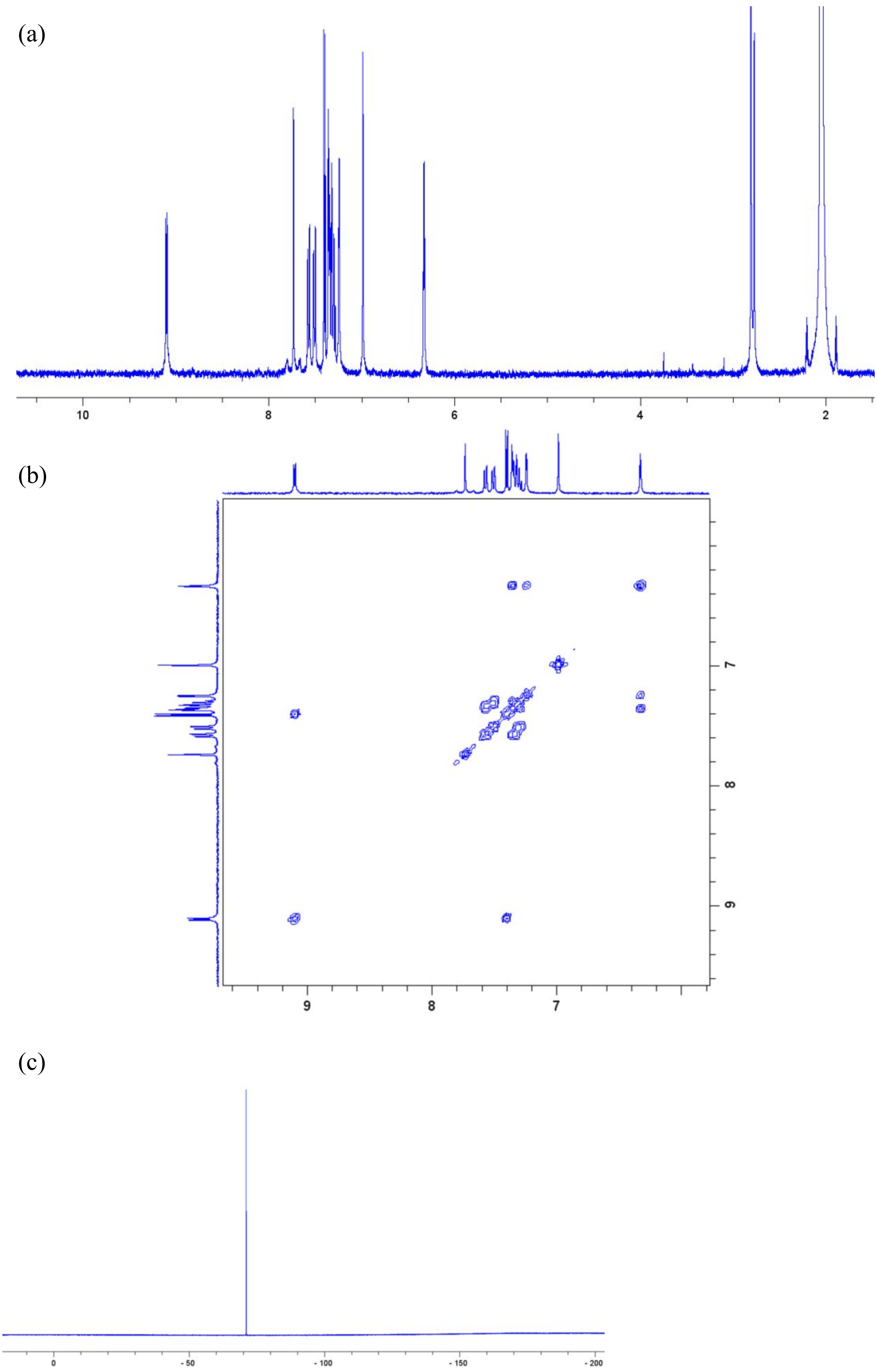

Figure S2. ^{1}H (a), ^{1}H-^{1}H COSY (b) and ^{19}F (c) NMR spectra of **2** in acetone-d_6.

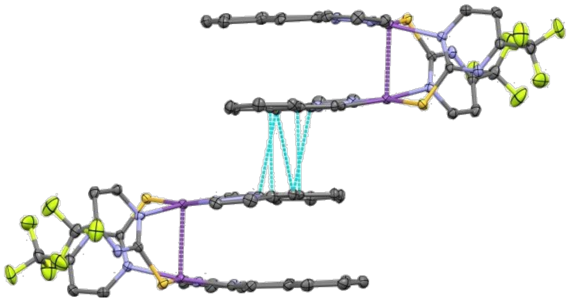

Figure S3. Crystal packing view of complex **2**

(a)

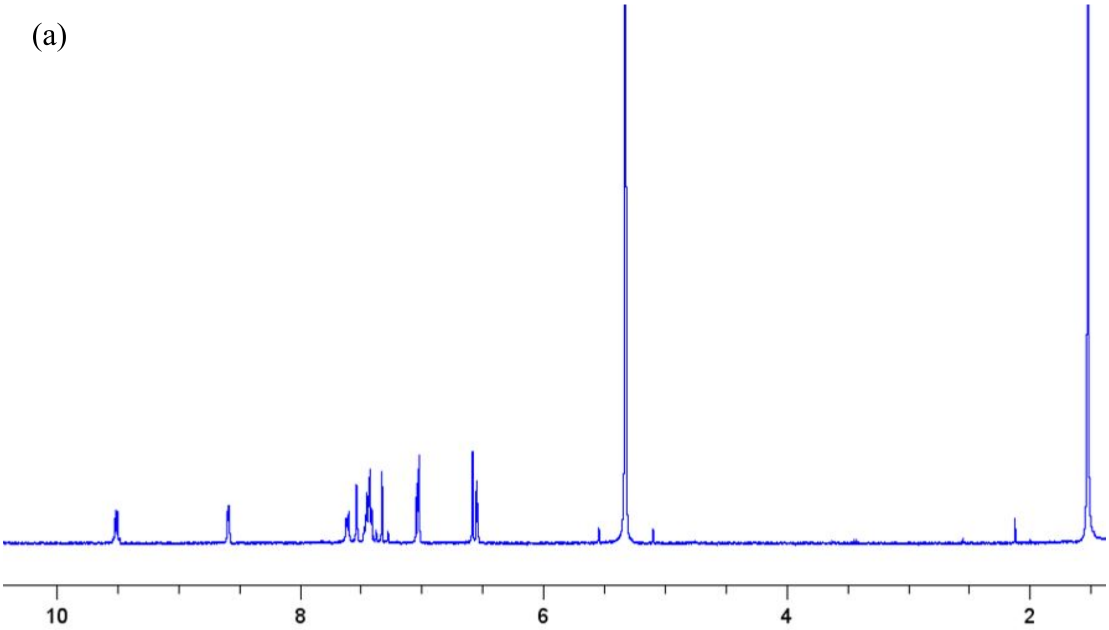

(b)

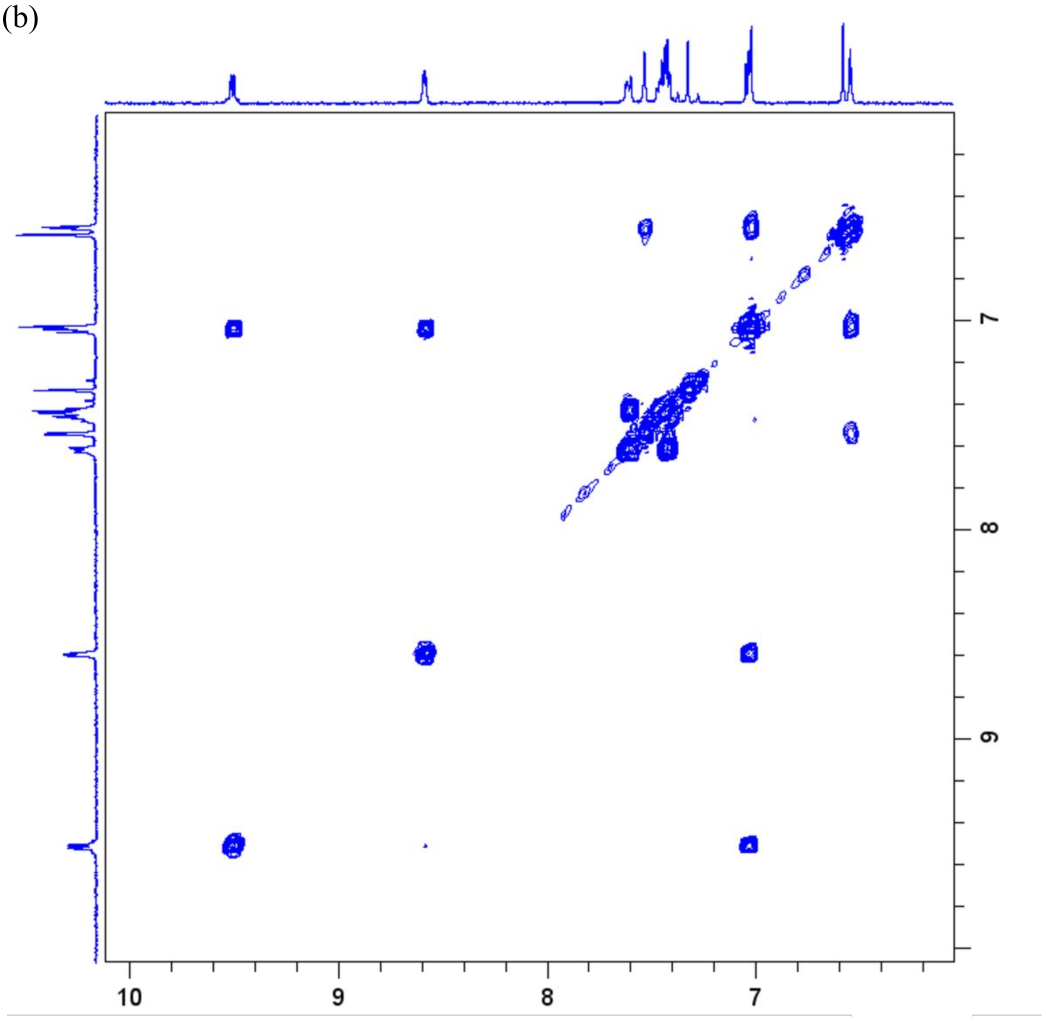

Figure S4. ^{1}H (a) and ^{1}H-^{1}H COSY (b) NMR spectra of **1-Cl** in CD_2Cl_2

(a)

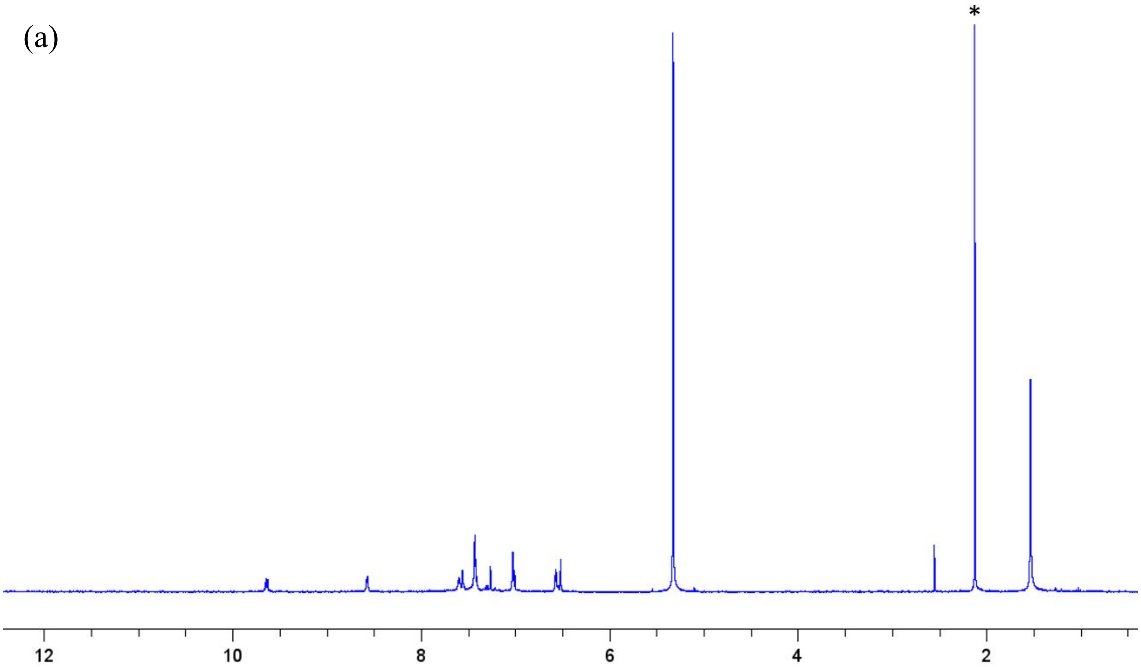

(b)

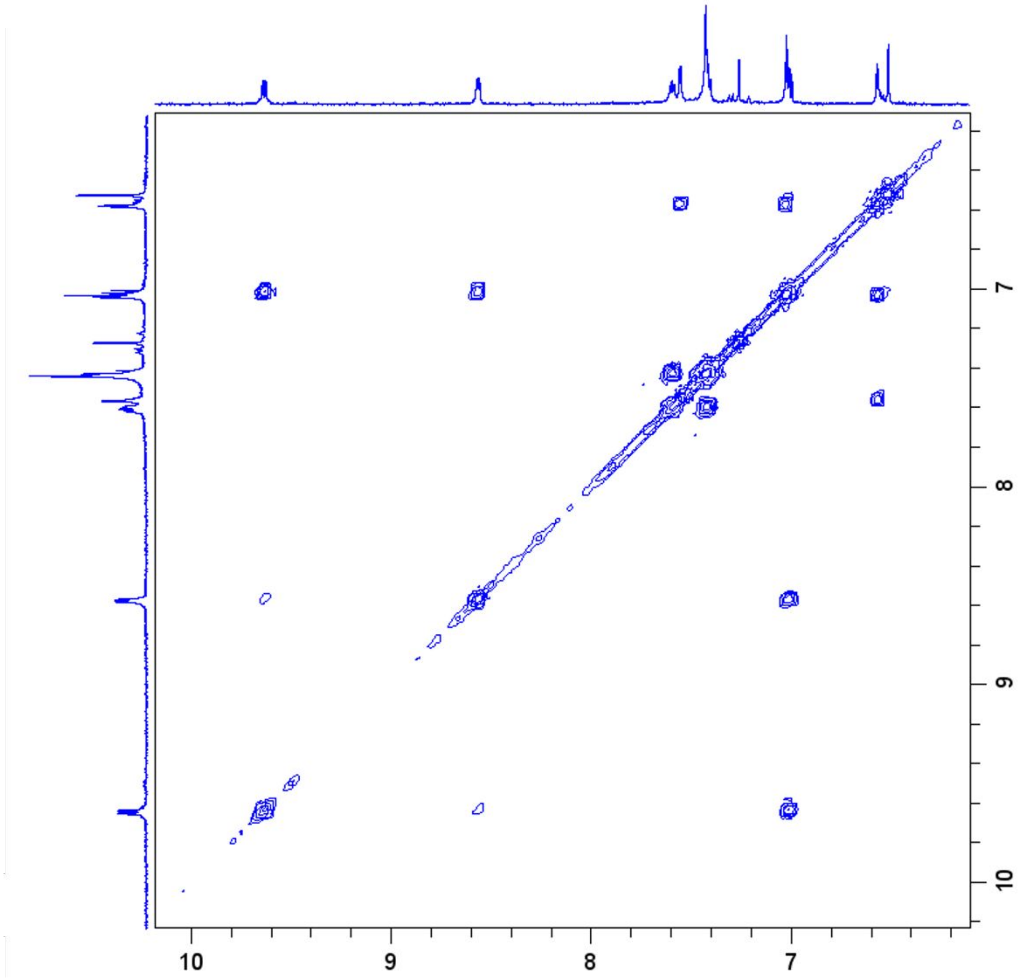

Figure S5. ^{1}H (a) and ^{1}H-^{1}H COSY (b) NMR spectra of **1-Br** in CD_2Cl_2 (* residual acetone)

(a)

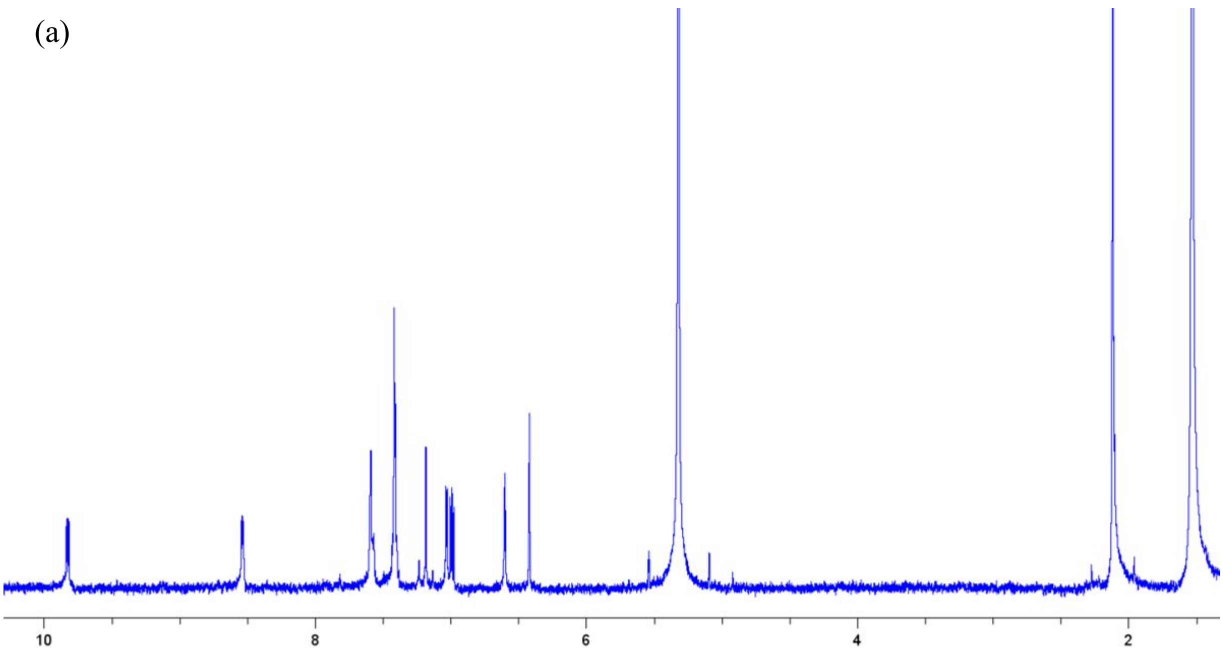

(b)

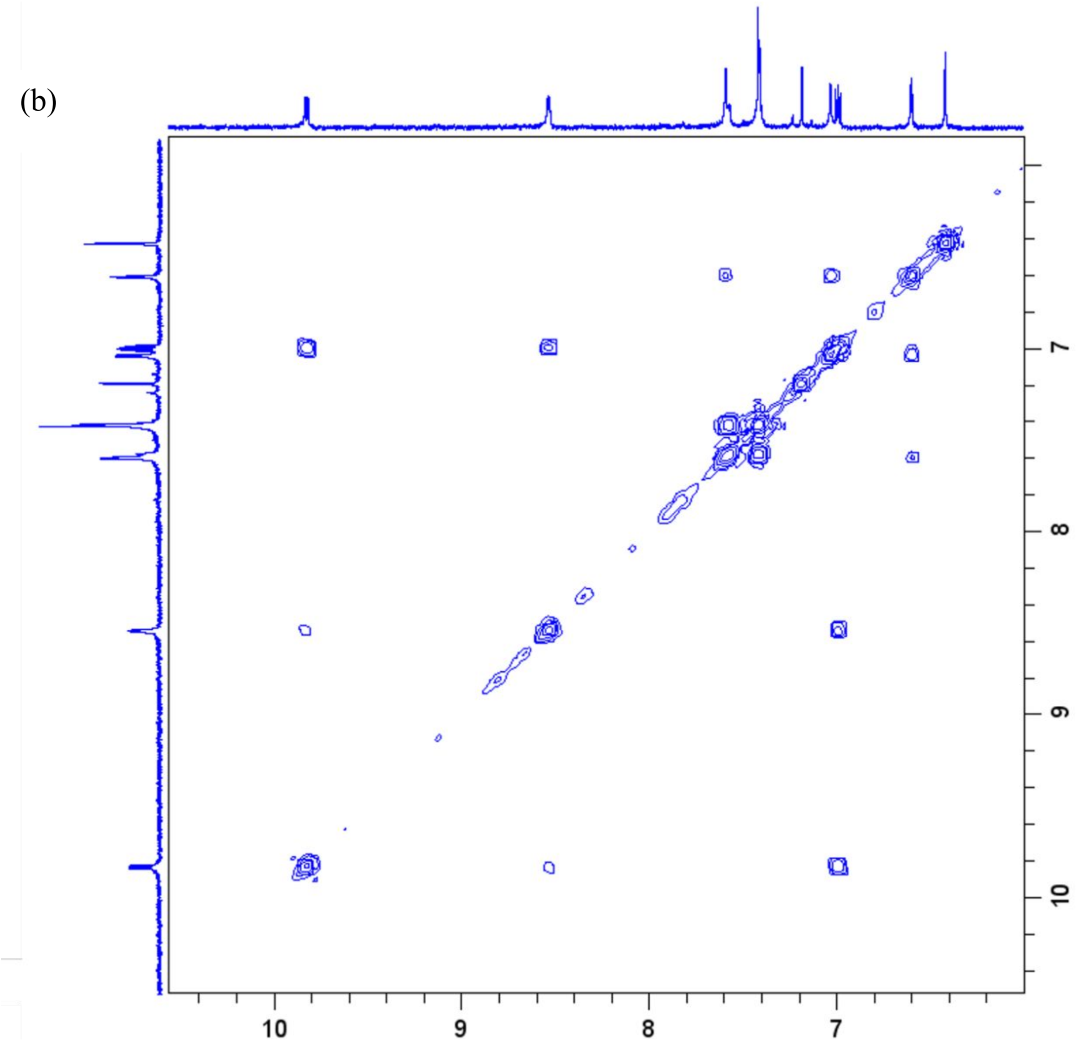

Figure S6. ^{1}H (a) and ^{1}H-^{1}H COSY (b) NMR spectra of **1-I** in CD_2Cl_2

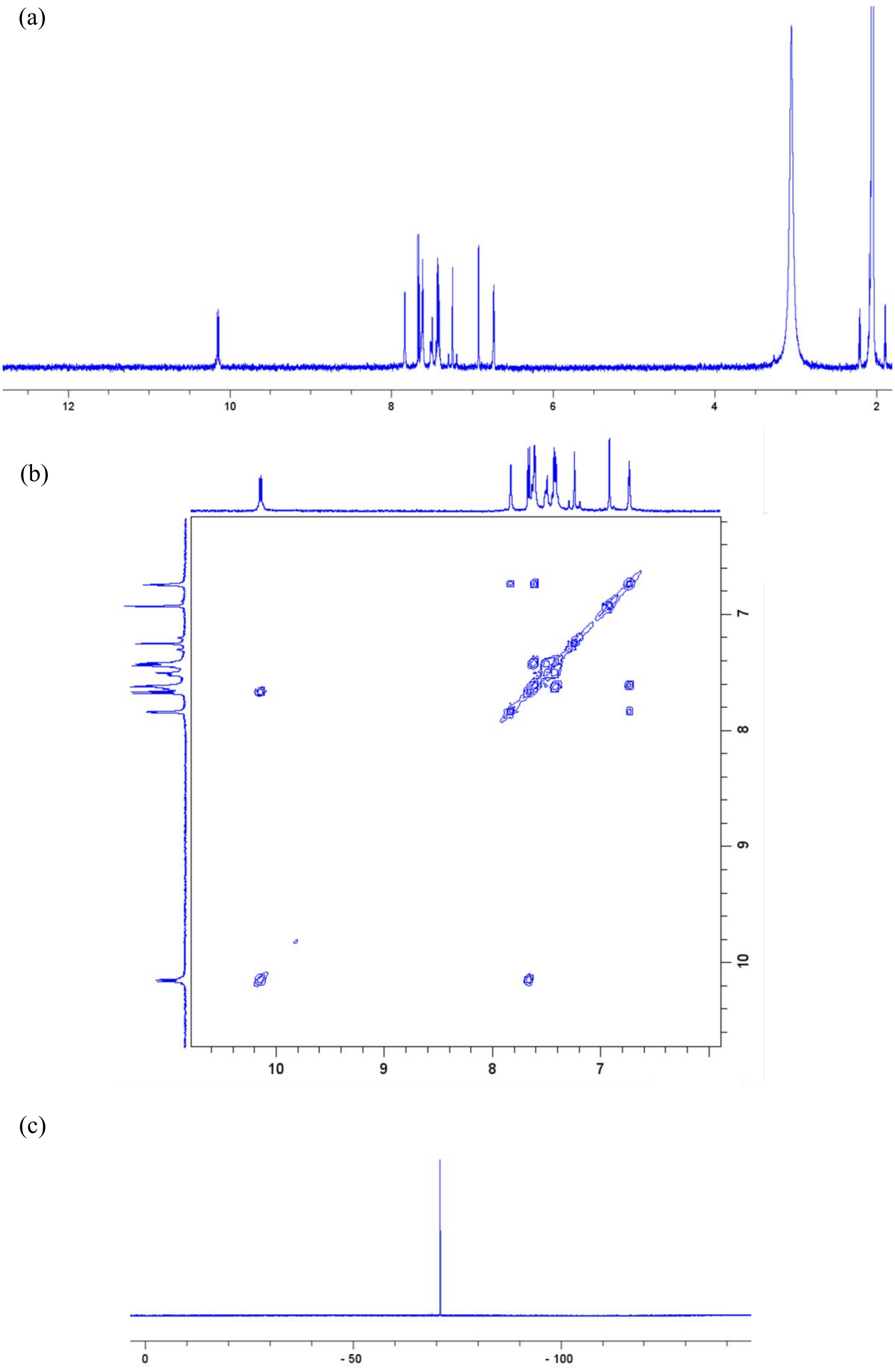

Figure S7. ^{1}H (a), ^{1}H-^{1}H COSY (b) and ^{19}F (c) NMR spectra of **2-I** in acetone-d_6

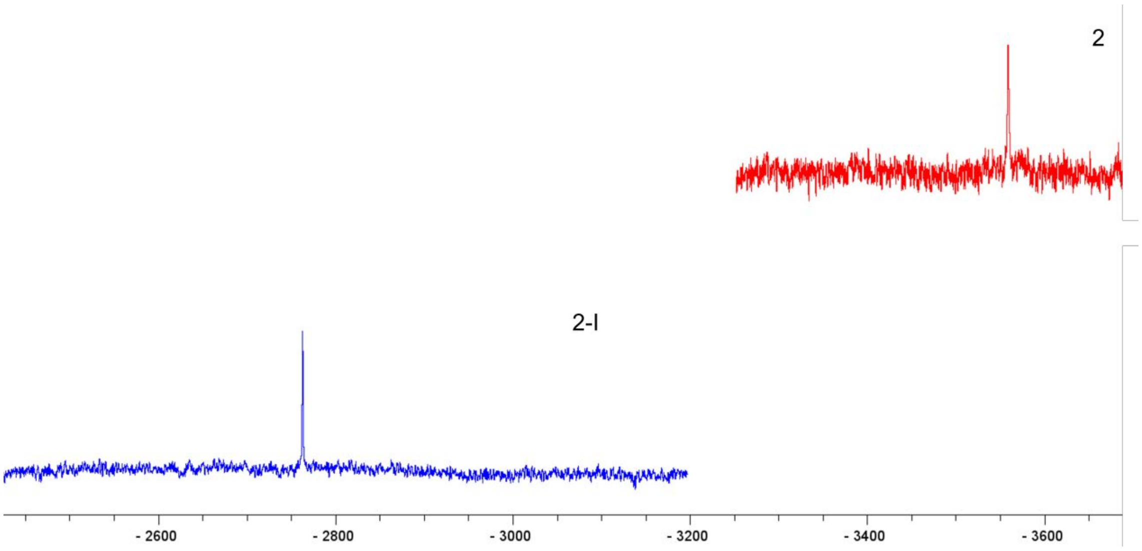

Figure S8. $^{195}Pt\{^{1}H\}$ NMR spectra of **2** and **2-I**

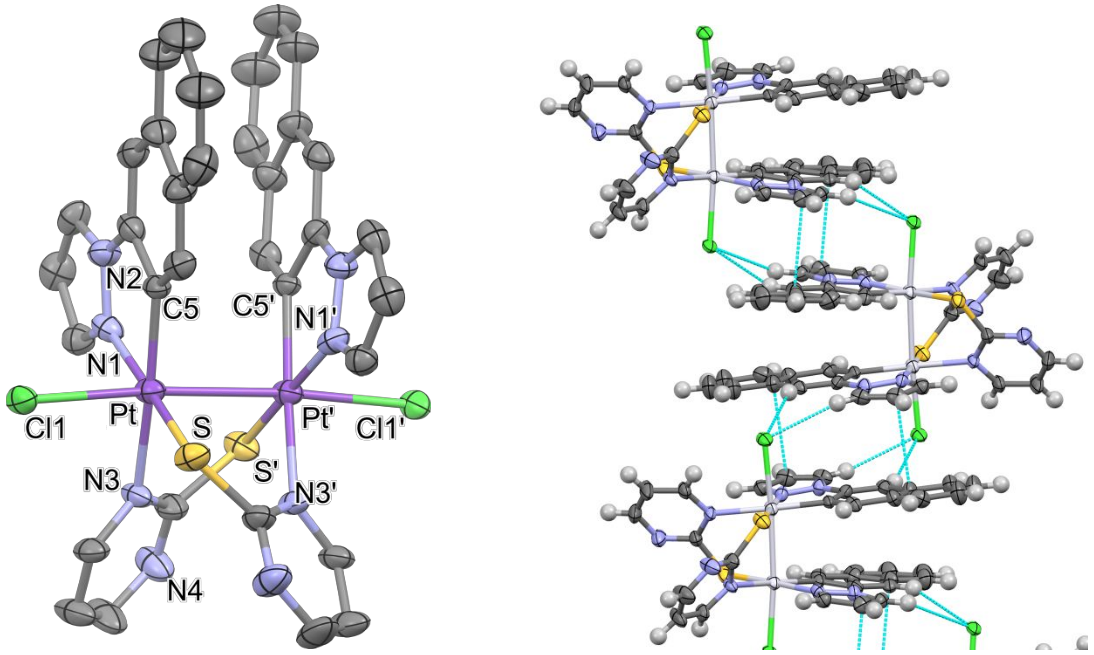

Figure S9. Left: Molecular structure of **1-Cl**. Thermal ellipsoids are drawn at their 50% probability level, solvent molecules and hydrogens are omitted for clarity. **Right**: crystal pacing of **1-Cl**.

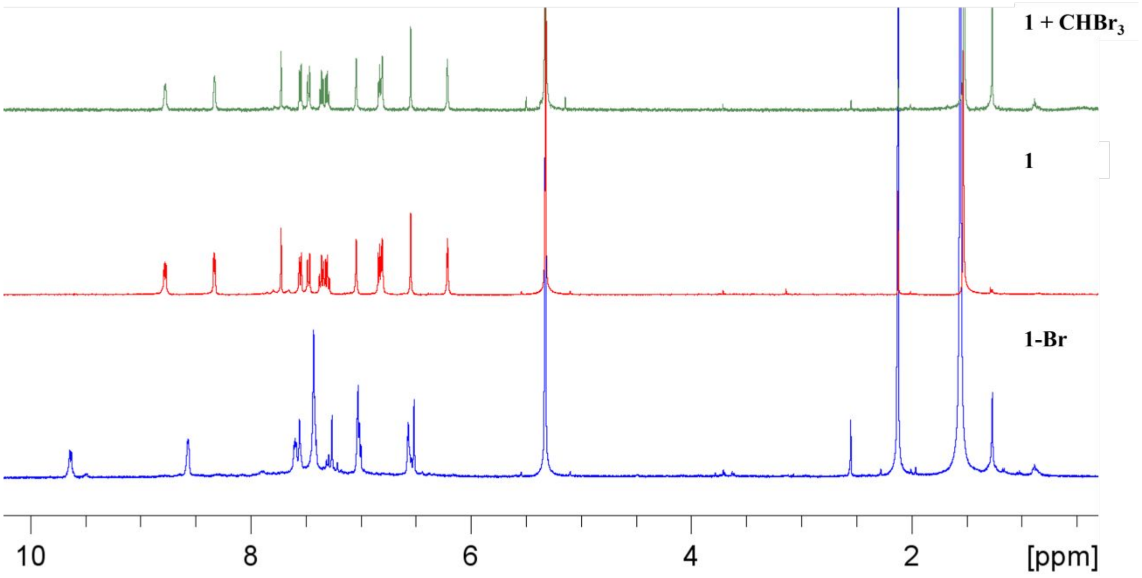

Figure S10. ^{1}H NMR spectra in CD_2Cl_2 of the resulting solid from the reaction of **1** with $CHBr_3$ (1:4) in acetone protected from light and in the air overnight (green line), **1** (red line) and **1-Br** (blue line).

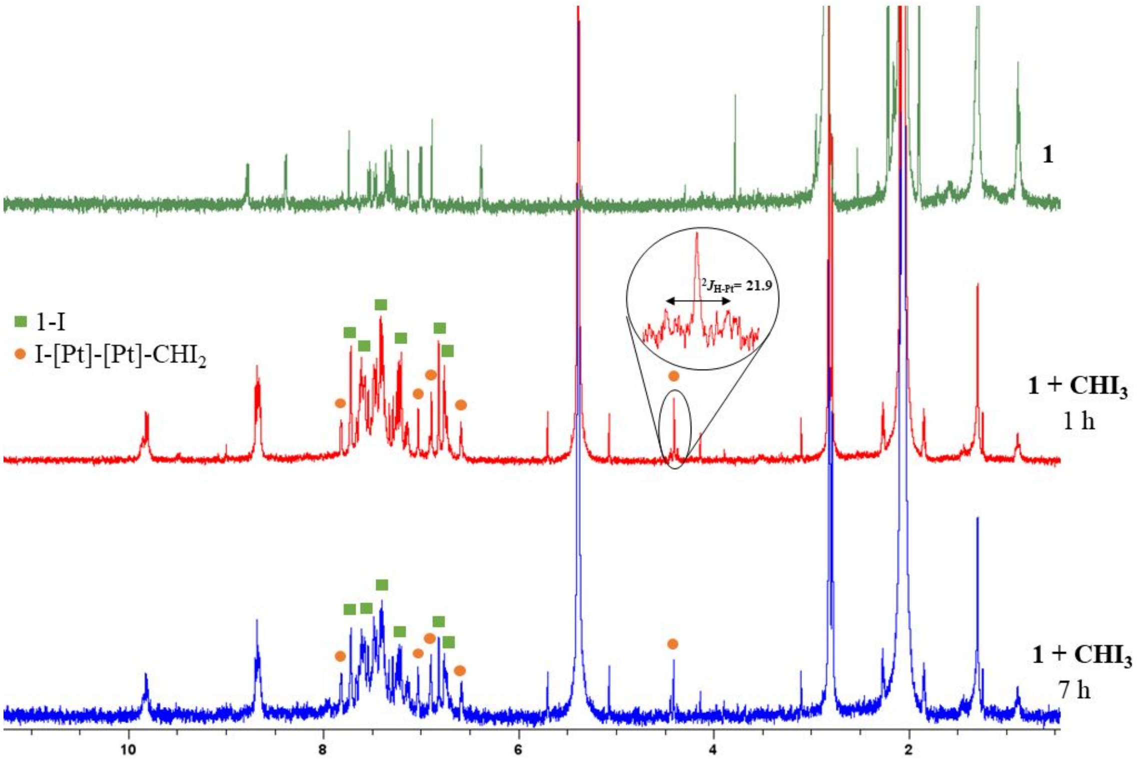

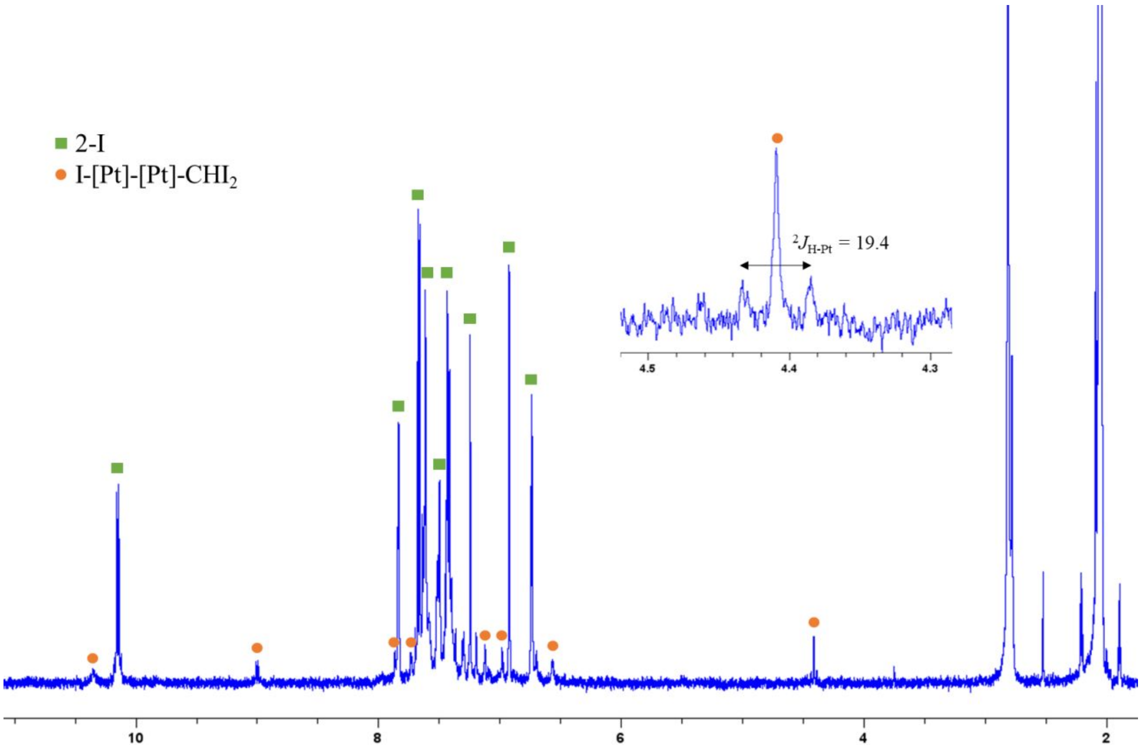

Figure S11. TOP: ^{1}H NMR spectra in acetone-d^6 of **1** (green) and **1** + CHI_3 (1:4) protected from light in the air at t=1hour (red) and t= 7 hour (blue). **BOTTOM:** ^{1}H NMR spectra in acetone-d^6 of the resulting solid from the reaction of **2** with CHI_3 (1:4) in acetone protected from light and in the air. Inset: Expanded view of the Pt-C*H*I_2 signal.

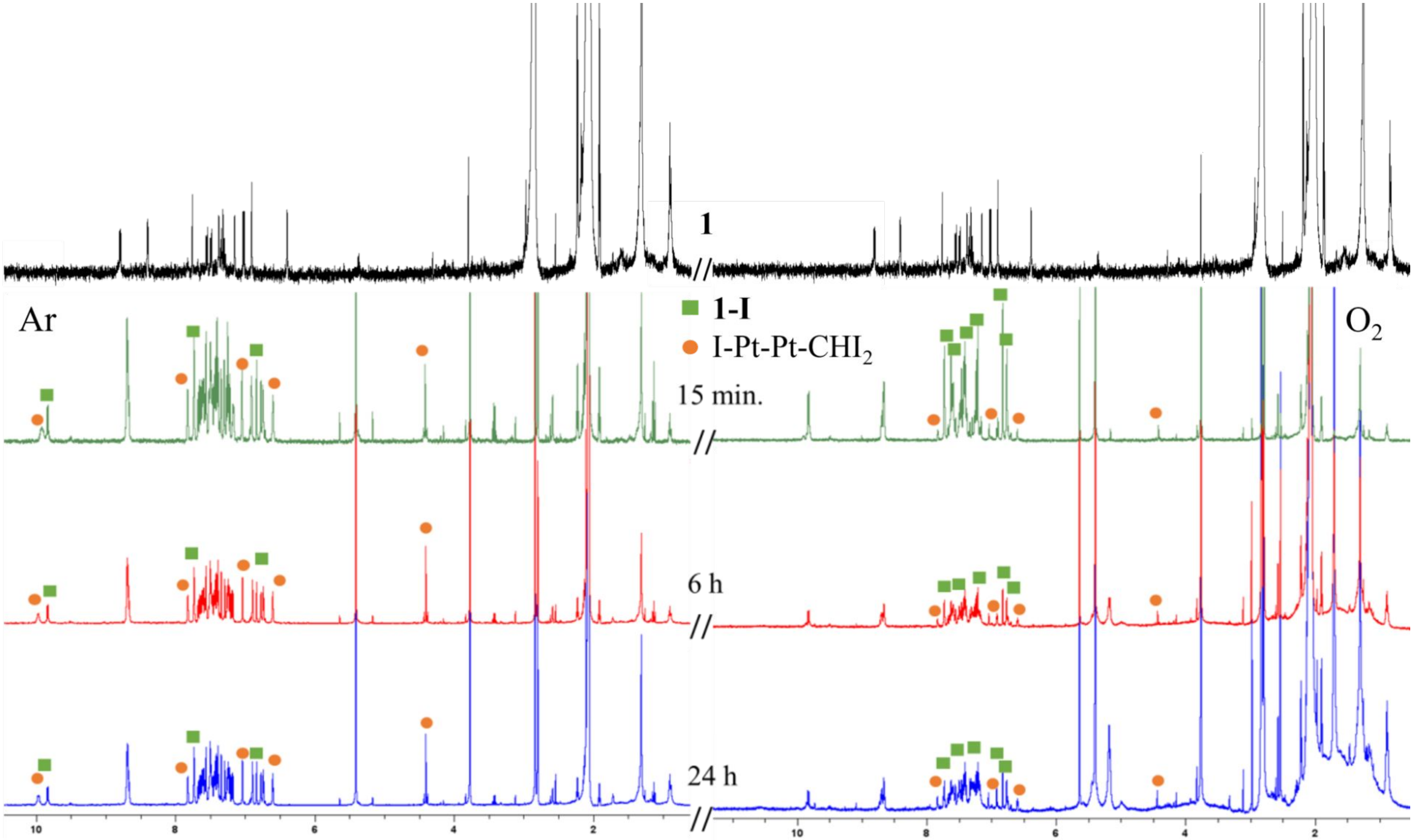

Figure S12. ^{1}H NMR spectra in acetone-*d*[6] of **1** (black line) and of **1** + CHI_3 (1:4) protected from light under Ar (right) and O_2 (left) atmosphere.
*Complex **1-I** precipitates in the reaction media

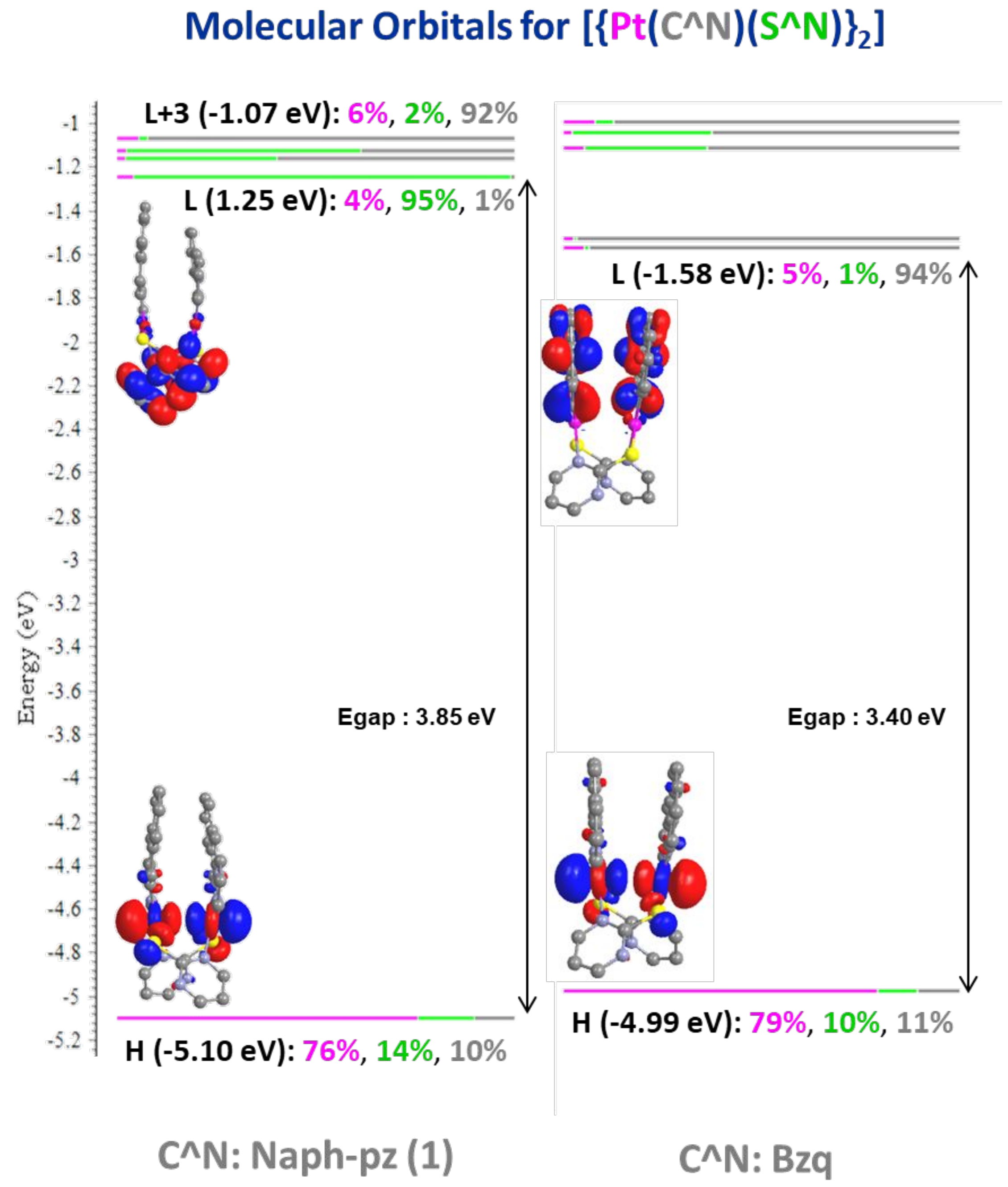

Figure S13. Schematic representation and composition of the frontier orbitals for complexes [{Pt(C^N)(μ-S^N)}$_2$] (HS^N: 2-mercaptopyrimidine; C^N: Naph-pz (left), Bzq (right)).

3. PHOTOPHYSICAL PROPERTIES AND THEORETICAL CALCULATIONS

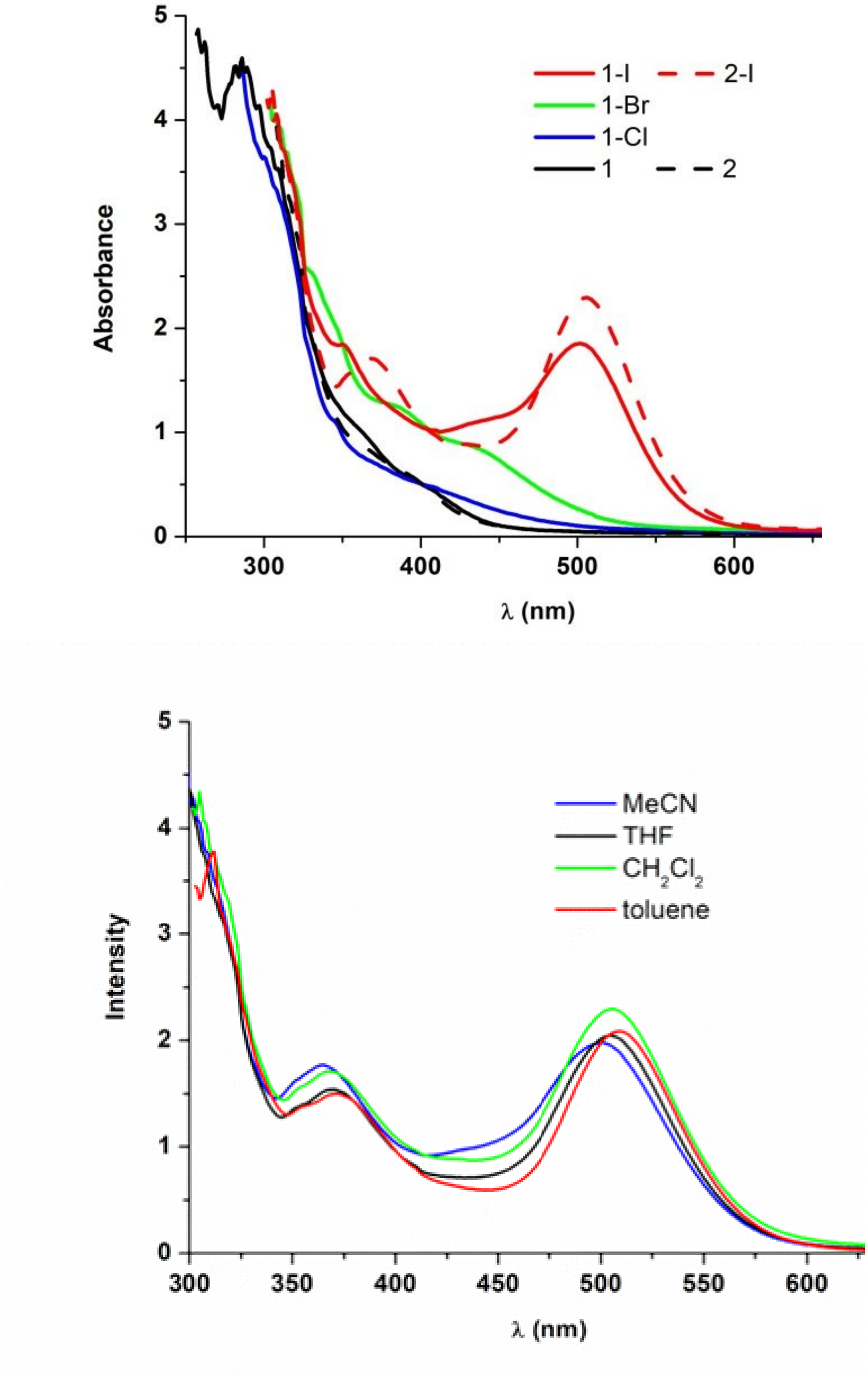

Figure S14. Absorption spectra of all complexes in CH_2Cl_2 solution at 10^{-4} M concentration (top) and absorption spectra of **2-I** in solution at 10^{-4} M (bottom).

Table S2. Population Analysis (%) of Frontier MOs in the Ground State for **1**, **2**, **1-Cl** and **1-I** in gas phase

MO	eV				Pt				C^N				S^N				X			
	**1**	**2**	**1-Cl**	**1-I**	**1**	**2**	**1-Cl**	**1-I**	**1**	**2**	**1-Cl**	**1-I**	**1**	**2**	**1-Cl**	**1-I**	**1**	**2**	**1-Cl**	**1-I**
L	-1.25	-2.08	-2.68	-2.97	4	3	52	44	1	1	7	6	95	96	16	12			25	38
H	-5.10	-5.35	-6.05	-5.64	76	73	16	18	10	16	29	13	14	11	22	10			33	59
H-2			-6.32	-6.08			5	6			70	2			22	1			3	91
H-3			-6.35				12				6				80				2	
H-5			-6.55				12				43				34				12	

Table S3. TD-DFT Vertical Excitations of Selected S_n excited states in gas phase

**Compound**	**λ[nm] (S_n)**	**o.s.**	**Transition (% contribution)***	**Assignment**
**1**	433.5 (S_1)	0.002	HOMO → LUMO (93%)	MM→L'CT
**2**	535.3 (S_1)	0.0066	HOMO → LUMO (97%)	MM→L'CT
**1-Cl**	505.4 (S_1)	0.0003	H-3 → LUMO (85)	
	498.1 (S_2)	0.0142	HOMO → LUMO (38%); H-5 → L (31); H-2 → L (18)	LL'→MMCT / LL'→XCT
**1-I**	601.8 (S_1)	0.0119	H-2 → LUMO (85); HOMO → LUMO (11)	X→MMCT

* Transitions with contributions < 10% were not included

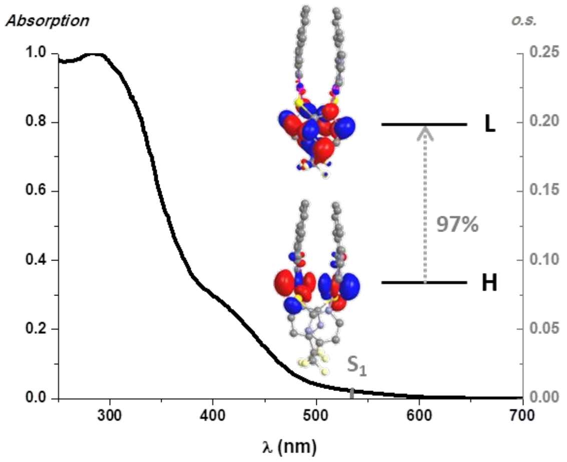

Figure S15. Normalized absorption spectra in solid state, calculated transitions in gas phase (grey bars) and molecular orbital plots (isovalue 0.03) for compound **2** (top).

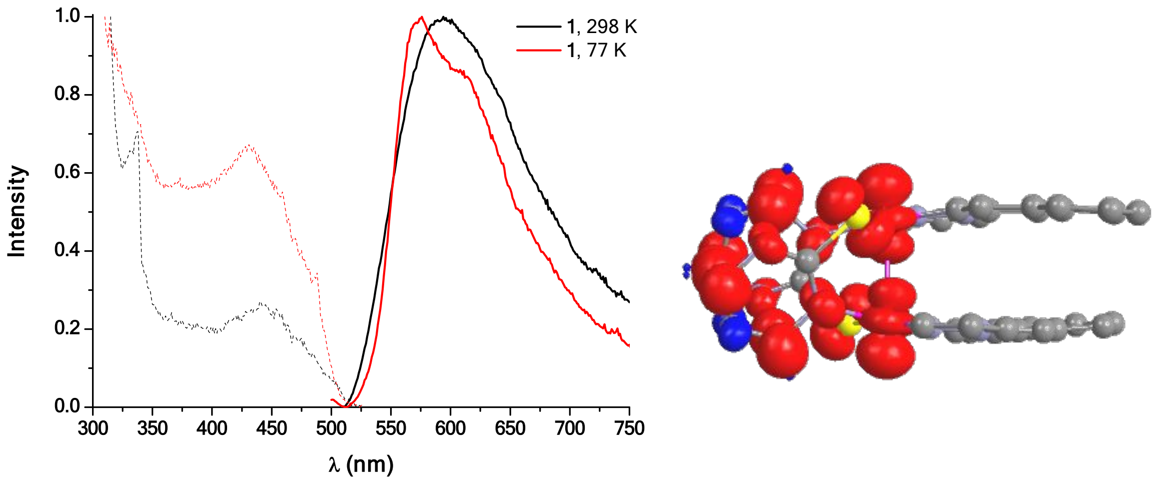

Figure S16. Left: Normalized excitation (dotted lines) and emission (solid lines) spectra of **1** in solid state. **Right**: Spin-density distribution of **1** calculated for the T_1 state (isovalue 0.003).

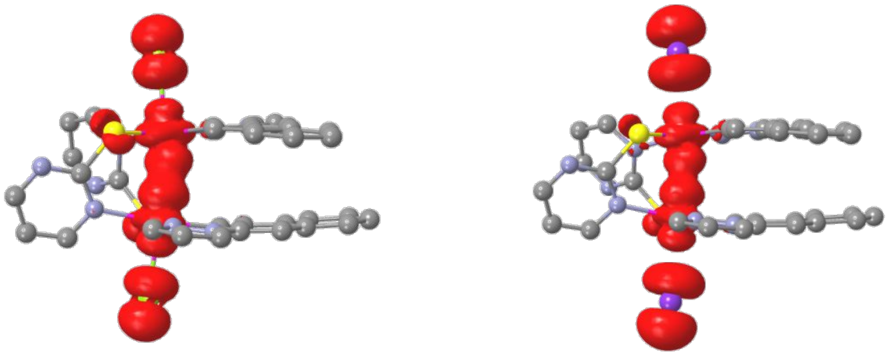

Figure S17. Spin-density distribution plots (isovalue 0.003) calculated in gas phase for the T_1 states.

Table S4. Calculated spin density distributions (%) in the first triplet state (T_1)

**Compound**	**Pt**	**C^N**	**S^N**	**X**
**1**	0.663	0.026	1.311	
**2**	0.675	0.033	1.292	
**1-Cl**	0.857	0.128	0.237	0.778
**1-I**	0.669	0.116	0.184	1.031

Table S5. Calculated bond parameters at the optimized geometries of the ground state (S_0) and the first triplet state (T_1)

	d Pt-Pt (Å) / Mayer BO	
**Compound**	**S_0**	**T_1**
**1**	2.964 / 0.27	2.820 / 0.51
**2**	3.006 / 0.25	2.825 / 0.52
**1-Cl**	2.675 / 0.61	2.988 / 0.27
**1-I**	2.714 / 0.57	2.949 / 0.26

4. REFERENCES

1. RED, C., CCD camera data reduction program *Rigaku Oxford Diffraction* **2019,** *Oxford Diffraction: Oxford, UK.*

2. Sheldrick, G. M., SHELXL Version 2014/8. *Acta Crystallogr. Sect. C* **2015,** *71*, 3-8.
